# Relationship of Healthy Building Determinants With Musculoskeletal Disorders of the Extremities: A Systematic Review

**DOI:** 10.7759/cureus.37456

**Published:** 2023-04-11

**Authors:** Ezequiel D Gherscovici, John M Mayer

**Affiliations:** 1 Research and Development, Healthy Buildings LLC, Malibu, USA

**Keywords:** extremities, musculoskeletal disorders, healthy buildings, indoor environmental quality, built environment

## Abstract

Musculoskeletal disorders (MSDs) are a substantial societal burden and various factors affect their causation, recovery, and prognosis. Management of MSDs is complex and requires multifaceted interventions. Given the challenges of MSDs and their continued burden, it is possible that additional elements could impact these disorders that have not been fully researched, for example, indoor environmental quality. Our previous review provided preliminary evidence that healthy building determinants (HBDs) are associated with the risk of back and neck pain. However, the relationship of HBDs with extremity MSDs and general MSDs (i.e., MSDs involving multiple body regions or in which body regions were unspecified in the original reports) has not been formally studied. The purpose of this review was to conduct a systematic literature review to assess the relationship of HBDs with extremity and general MSDs (PROSPERO ID: CRD42022314832). PubMed, CINAHL, Embase, and PEDRo databases were searched through April 2022. Inclusion criteria for study eligibility were as follows: humans of ages ≥18 years, reported on one or more of eight HBDs (1. air quality and ventilation, 2. dust and pests, 3. lighting and views, 4. moisture, 5. noise, 6. safety and security, 7. thermal health, 8. water quality), and compared these HBDs with extremity MSDs or general MSDs, original research, English. Exclusion criteria were as follows: articles not published in peer-reviewed journals, full-text articles unavailable. Review procedures were conducted and reported in accordance with Preferred Reporting Items for Systematic Reviews and Meta-Analyses (PRISMA) recommendations. Empirical evidence statements were developed for 33 pairwise comparisons of HBDs with MSDs. The search uncovered 53 eligible studies with 178,532 participants. A total of 74.6% (39/53) of the studies were cross-sectional and 81.1% (43/53) were fair quality. Overall, the majority of uncovered evidence indicates that HBDs are related to risk of extremity and general MSDs. Nineteen comparisons support that as HBDs worsen, the risk of MSDs increases. Six comparisons had conflicting evidence. Three comparisons support that poor HBDs are not related to increased risk of extremity and general MSDs. Five comparisons had no evidence. This systematic review builds upon previous work to provide useful starting points to enhance awareness about the HBD-MSD relationship. These findings can help inform research and public health efforts aimed at addressing suboptimal HBDs through appropriate interventions to improve the lives of those suffering from MSDs.

## Introduction and background

Musculoskeletal disorders (MSDs) are common, significantly impact the quality of life and physical abilities of individual sufferers, and are a substantial burden on society [1,2]. While numerous clinical, policy, and environmental approaches have been effective to attenuate many chronic diseases [3], chronic MSDs remain more troublesome in terms of disability than some chronic internal diseases of greater morbidity and mortality [4]. For example, many of the top conditions leading to years lived with disability (YLDs) are MSDs including low back pain (#1), neck pain (#6), other MSDs (#7), falls (#10), and osteoarthritis (#12), while some of the major internal diseases rank lower, such as diabetes (#8), chronic obstructive pulmonary disease (#11), ischemic stroke (#17), and ischemic heart disease (#29) [4].

Musculoskeletal disorders involve various connective tissues, such as bones, joints, muscles, tendons, and ligaments [2], across several body regions, such as the spine (e.g., neck, back), extremities (e.g., arms, legs), and general body regions (e.g., some types of arthritis) [1]. The distinction between body regions affected by MSDs is clinically important, particularly for spinal versus extremity MSDs, since these MSDs are different entities and may have different etiologies, risk factors, and prognoses. Moreover, the recommended interventions for spinal versus extremity MSDs may be different, requiring management by separate medical sub-specialties [5-7].

Various factors affect the causation, recovery, and prognosis of MSDs, and MSDs are causal agents themselves [8-12]. Furthermore, multi-faceted interventions are required to manage MSDs including medications, exercises, psychosocial interventions, bodyweight and general health guidance, and possibly surgical interventions [8-12]. Given the complexities and challenges of MSDs and their ongoing burden, it is possible that additional elements could impact these disorders that have not been fully researched, for example, indoor environmental quality [13]. A 2001 survey of people in the United States found that approximately 90% of people's time is allocated to the indoor built environment [14,15]. Therefore, the health sequelae of being indoors are worth investigating, with the interest here focused on extremity MSDs [13].

The concept of healthy buildings is a "biopsychosocial framework that focuses on transforming the built environment to promote and enhance the health, wellness, performance, productivity, and quality of life of occupants," as we previously defined [13]. The World Health Organization indicates that a healthy building is "a space that supports the physical, psychological, and social health and well-being of people" [16]. Several healthy building determinants (HBDs) have been discussed over the past two decades, including these eight general categories: (1) air quality and ventilation, (2) dust and pests, (3) lighting and views, (4) moisture, (5) noise, (6) safety and security, (7) thermal health, and (8) water quality [15,17,18]. Even though there is lack of standards for characterizing components of HBDs, these eight HBDs are starting points for future research, policy, and practice efforts.

It is plausible that addressing HBDs within the indoor built environment could be useful for addressing MSDs [13]. Furthermore, expanding the understanding of the HBD-MSD relationship could impact stakeholders in healthcare, real estate, occupational, policy, and public health sectors, and may result in interventions to enhance the quality of life, performance, and productivity of people with MSDs in various indoor built environment settings (e.g., residential, commercial, occupational, and public) [13]. However, the HBD-MSD relationship has not been comprehensively investigated and the extent of available interventions for this human-environment-building interface is unclear.

Our 2022 systematic review examined the relationship of HBDs with spinal MSDs [13], which provided preliminary evidence that HBDs are associated with the risk of back and neck pain. However, the relationship of HBDs with extremity MSDs and general MSDs (i.e., MSDs involving multiple body regions or in which body regions were unspecified in the original reports) has not been formally studied. Additionally, healthy building reports, built environment regulations, and clinical practice guidelines do not sufficiently address this association. Therefore, the purpose of this review was to conduct a systematic literature review to assess the relationship of HBDs with extremity MSDs and general MSDs.

## Review

Materials and methods

Overview

The current review incorporated similar methods, evidence synthesis procedures, and reporting structure as our earlier systematic review that examined HBDs and spinal MSDs [13]. Taken together, these companion studies provide a comprehensive assessment of the HBD-MSD relationship. The current review was carried out and reported based on the Preferred Reporting Items for Systematic Reviews and Meta-Analyses (PRISMA) [19], and other resources [8,20-26]. The review was registered with PROSPERO (ID: CRD42022314832).

Information Sources

The search approach was adapted from our earlier systematic review that examined HBDs and spinal MSDs [13]. Studies were uncovered by searching PubMed, CINAHL, Embase, and PEDRo. The last author (JM) developed the search strategy and the first author (EG) cross-checked it. The PubMed search strategy is shown in the Appendices, and CINAHL, Embase, and PEDRo were searched using a comparable database-specific approach. Hand searches of authors' files and examination of references within studies obtained from the primary search were carried out to identify additional studies.

Eligibility Criteria

Inclusion and exclusion criteria are depicted using the PICOTS method [13,19].

P - patient/people: Studies were included if they assessed humans of the ages 18 years and older with MSDs involving the extremity body regions or general body regions (i.e., MSDs involving multiple body regions or in which body regions were unspecified in the original reports). The definition of MSDs is described elsewhere [1,13]. Upper extremity (upper limb) is the region of the upper body extending from the shoulder (proximally) to the fingers (distally), including the shoulder, upper arm, elbow, forearm, wrist, hand, and fingers [27]. Lower extremity (lower limb) is the region of the lower body extending from the hip (proximally) to the toes (distally), including the hip, thigh, knee, leg, ankle, foot, and toes [28]. Studies were included that described all types, severities, and chronicities of MSDs, and were excluded if they only described systemic disorders, such as fibromyalgia, or neurological conditions, such as multiple sclerosis [13].

I - intervention: Studies were included if they examined HBDs within the following eight categories: (1) air quality and ventilation, (2) dust and pests, (3) lighting and views, (4) moisture, (5) noise, (6) safety and security, (7) thermal health, and (8) water quality [18]. While other components of HBDs are possible, these eight HBDs were selected because they have been previously reported and can provide starting points for future research, policy, and practice efforts [18]. The HBD category of "thermal health" was divided into three sub-categories (uncomfortable, cold, warm) since the studies reported on cold, warm, or unspecified thermal health separately. The sub-category of "thermal health - uncomfortable" was included since some studies presented the thermal health outcome without specifying whether it was related to warm or cold indoor environments. Also, authors of some studies presented several HBD categories within one outcome measure. Thus, for the purpose of the current review, we created a new HBD variable named "overall work environment," which is an aggregate variable consisting of several HBD categories.

Pertinent definitions for this review are found elsewhere for healthy buildings [13,16], built environment [29], determinants of health [30], and HBDs [13]. Except for case reports, all other types of subject-level original research studies were eligible for inclusion.

C - comparator: Studies were eligible for inclusion if they compared the previously described HBDs with extremity or general MSDs (i.e., MSDs involving multiple body regions or in which body regions were unspecified in the original reports). The independent impact of the HBD on the MSD must have been assessed.

O - outcomes/variables: Studies were included if they used various strategies to assess HBDs and MSDs, such as patient-reported, physical, functional, and environmental outcome measures. Studies were included that examined measures directly associated with MSDs, for example, disability and lost work time, while those that only examined outcomes indirectly related to MSDs, such as body mass, lifestyle, and psychosocial measures, were excluded [13].

T - time/timing: Studies were included if they were published in peer-reviewed journals from database inception through April 15, 2022.

S - setting: Studies were included if they assessed the previously described HBDs within the indoor built environment of commercial, public, residential, or work-related real estate settings. Studies were excluded that assessed outdoor environments [13].

Additionally, studies were included if they were published in a peer-reviewed journal in English, human research, original research at the subject level, abstract was available for preliminary screening, and full-text article was available for the final determination processes. Studies were excluded if they were non-human studies (e.g., animal, basic science, laboratory, or simulation research), or grey literature, case reports, or reviews [13]. Studies were also deemed ineligible if they assessed ergonomic factors, such as safe patient handling, lifting, materials handling, and worksite vibration. Ergonomic factors were excluded because they have been examined in other literature and they are not underscored in the previously reported foundations for healthy buildings [18,31].

Data Extraction

Study selection: Search results were handled using citation manager and spreadsheet databases [13]. After preliminary management of the extracted articles, EG and JM separately screened citation information (e.g., title, abstract) of the extracted articles to assess eligibility. Articles were classified as relevant, possibly relevant, or irrelevant. Subsequent to reaching a consensus, full-text PDFs were acquired for articles considered to be relevant or possibly relevant. EG and JM separately screened the full-text articles for relevance. Then, the two authors worked together to reach a consensus on the final eligible articles. Automation was not utilized in the process of selecting studies [13].

Data extraction: JM extracted data from the eligible articles and entered them into the database, and EG separately cross-checked the results [13]. Then, the two authors worked together until reaching a consensus regarding the extracted data. Automation was not utilized for the data extraction processes. Extracted data entered in tables were - author, year, country, funding source, population, sample size, gender, age, inclusion and exclusion criteria, which HBD was examined, which HBD outcome measures were used, MSD type (upper extremity, lower extremity, general), which MSD outcome measures were used, analysis procedures, and results. Missing data were not considered in the evidence synthesis procedures and are reported in the tables. If needed, authors of the eligible articles were contacted by email to elucidate study findings [13].

Outcome measures: Since the uncovered evidence was mostly obtained from observational studies, the outcome variables measured in the eligible articles were mostly descriptive and relational [13].

Data Synthesis

Approaches adapted from the Oxford Centre for Evidence-Based Medicine, Clinical Information Access Portal [20-23], and American Physical Therapy Association [8,24] were used to handle data and synthesize the evidence [13].

Study quality and level of evidence: Study quality (risk of bias) was assessed using the NIH quality assessment instrument for observational studies [32]. The instrument has 14 items in which an item is rated as yes = 1, or no = 0 (total instrument score is 0-14) [32]. The authors calculated score ranges for study quality categories [32] from the total score [13]: 0-4 = poor quality (high risk of bias), 5-9 = fair quality (between low risk and high risk of bias), 10-14 = good quality (low risk of bias) [32]. Level of evidence (study type) was categorized using approaches adapted from the Oxford Centre for Evidence-Based Medicine [20-23]. JM assessed study quality and evidence level and EG separately cross-checked the results. Subsequently, the two authors worked together until reaching a consensus about study quality and evidence level. Automation was not utilized in the processes to assess study quality and evidence level. Reporting bias was not formally assessed and the tables report missing data [13].

Evidence synthesis: Empirical evidence statements were synthesized based on strategies adapted from the Oxford Centre for Evidence-Based Medicine [21-23], American Physical Therapy Association [8,24], and relevant systematic reviews [13,26]. Empirical evidence statements were based on pairwise comparisons with each assessing one MSD region by one HBD category (including the three thermal health subdivisions and the aggregate variable of overall work environment). Thus, 33 pairwise comparisons were assessed: three MSD regions (upper extremity, lower extremity, general) by 11 HBD categories and subdivisions (1. air quality and ventilation, 2. dust and pests, 3. lighting and views, 4. moisture, 5. noise, 6. safety and security, 7. thermal health -uncomfortable, 8. thermal health - cold, 9. thermal health - warm, 10. water quality, and 11. overall work environment). Empirical evidence statements for the pairwise comparisons were constructed with the following categories [8], which were also used in our previous review [13]: strong evidence - "one or more level I systematic reviews support the recommendation" [8], moderate evidence - "one or more level II systematic reviews or a preponderance of level III systematic reviews or studies support the recommendation" [8], weak evidence - "one or more level III systematic reviews or a preponderance of level IV evidence supports the recommendation [8], conflicting evidence - "higher-quality studies conducted on this topic disagree with respect to their conclusions and effect" [8], or no evidence. Considering this systematic review's broad purpose and the evidence uncovered, meta-, heterogeneity-, and sensitivity analyses were not carried out.

Results

Study Selection

**Figure 1:**
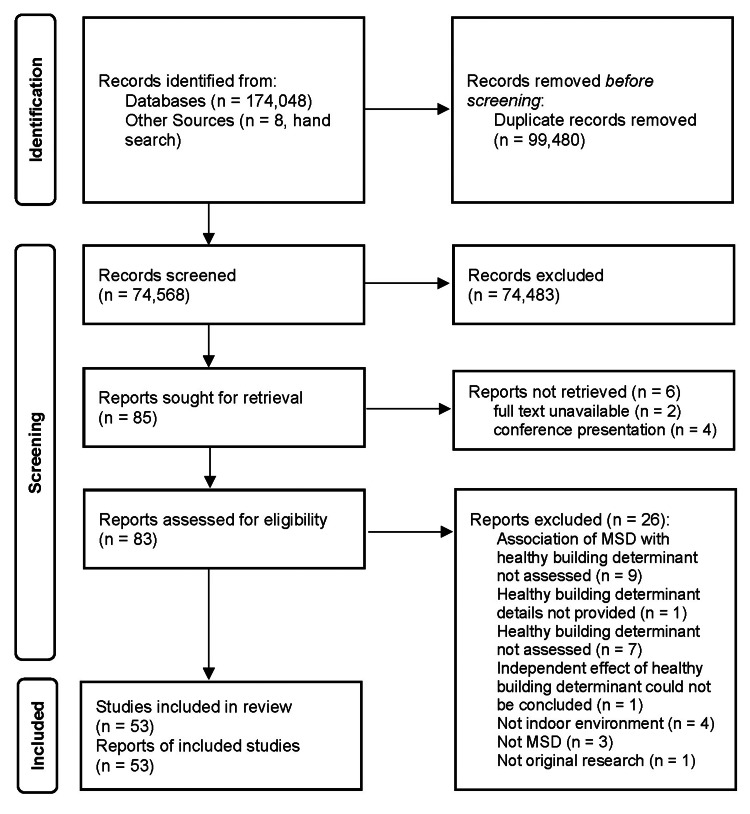
PRISMA diagram depicting search results. PRISMA: Preferred Reporting Items for Systematic Reviews and Meta-Analyses

Search results are found in the PRISMA diagram (Figure 1). Fifty-three eligible studies were uncovered [33-85]. Twenty-six studies seemed to be eligible at preliminary review, but were deemed ineligible for the following reasons: association of MSD with HBD not assessed (n = 9) [86-94], HBD details not provided (n = 1) [95], HBD not assessed (n = 7) [96-102], independent impact of HBD could not be concluded (n = 1) [103], not indoor environment (n = 4) [104-107], not MSD (n = 3) [108-110], and not original research (n = 1) [111].

Sixteen studies [34,38,47-50,58,63,67,69,70,72,73,75,78,79] uncovered in the current review were also reported in our other systematic review on HBDs and spine-related MSDs (back pain, neck pain) [13]. Of these 16 studies, 11 studies presented separate data for extremity/general MSDs and spine-related MSDs [47-50,63,67,70,72,75,78]. Eight studies presented data for MSD variables that could not be distinguished between extremity/general MSDs and spine-related MSDs and thus were included in both systematic reviews, including neck-shoulder pain [34,58,72,79], musculoskeletal pain across several regions [38,69], back pain/joint pain [73], and back pain/muscular pain [78].

Study Characteristics

Details of study characteristics and outcomes are shown in the Appendices. Overall, 178,532 individuals participated in the 53 included studies. Most studies (35/53) assessed workers in various occupational settings [33-38,45-51,54,55,57-62,65-68,71-75,77-80,82]. Three studies assessed the general population in residential settings [52,63,64], one of these assessed residential dwelling type [52]. One study assessed workers in both occupational and residential settings [53]. Fourteen studies assessed the general population in unstated settings [39-44,56,69,70,76,81,83-85]. Four studies included race and ethnicity in the statistical models for the HBD-MSD relationship [52,60,64,81], yet only one study assessed the independent effect of race and ethnicity on this relationship [81].

Upper extremity MSDs were assessed in 32 studies [34,36,37,39-42,44-52,54,57-59,61,67,70-72,74-77,79,80,84], with regions as follows: shoulder (n = 21) [34,36,40-42,45,47,48,50-52,58,61,67,70-72,74,75,79,80], arm (n = 4) [47,49,50,52], elbow (n = 11) [36,39,45,48-51,59,67,70,71], forearm (n = 2) [44,70], wrist/hand/fingers (n = 16) [36,45,47-52,59,67,70-72,75,76,84], and unspecified upper extremity region (n = 6) [37,46,54,57,76,77]. Lower extremity MSDs were assessed in 19 studies [36,37,43,46-48,50,52,53,56,61-64,66,67,70,71,85], with regions as follows: hip (n = 9) [43,47,48,52,53,56,66,67,71], thigh (n = 4) [48,67,70,71], knee (n = 12) [48,50,52,53,61-64,66,67,70,71], leg (n = 6) [36,47,52,61,63,70], ankle/foot/toes (n = 7) [47,48,50,66,67,70,71], and unspecified lower extremity region (n = 3) [37,46,85]. General unspecified MSDs were assessed in 14 studies [33,35,38,55,60,65,68-70,73,78,81-83].

Various HBDs were assessed in the included studies as follows: air quality and ventilation (N = 16) [33,38,49,51,53-55,57,64-66,69,71,77,79,83], dust and pests (n = 5) [33,57,62,71,77], lighting and views (n = 15) [34,45,49,51,54,57,58,61,62,65,66,72,74,77,79], moisture (n = 3) [46,54,79], noise (n = 15) [34,35,45,49,51,52,54,57,62,65,66,72,74,77,79], safety and security (n = 3) [60,80,82], thermal health (n = 32) [34,36,37,39-42,46-48,50,51,54,57,59,62,65-68,70,72,74-79,81,83-85], water quality (n = 5) [43,44,56,63,73], and overall work environment that was comprised of several HBDs (n = 4) [54,65,77,79].

The studies were conducted in numerous countries as follows: Australia (n = 2) [39,81], Australia and New Zealand (n = 1) [34], Bangladesh (n = 1) [33], Brazil (n = 3) [35,37,72], Canada (n = 1) [38], China (n = 3) [49,82,83], Colombia (n = 1) [67], Denmark (n = 3) [52,69,77], Ethiopia (n = 1) [58], Finland (n = 4) [56,59,70,75], India (n = 7) [55,61,62,65,71,73,74], Iran (n = 1) [64], Israel (n = 1) [54], Italy (n = 3) [45,57,80], Japan (n = 1) [50], Norway (n = 6) [36,43,44,47,79,85], Pakistan (n = 1) [48], South Korea (n = 1) [53], Sweden (n = 2) [76,84], Thailand (n = 4) [51,63,66,78], Turkey (n = 1) [68], and United States (n = 5) [40-42,46,60]. The publication years of the studies ranged from 1981 through 2021 as follows: 1980-1989 (n = 1) [38], 1990-1999 (n = 3) [49,56,85], 2000-2009 (n = 7) [36,55,67,70,72,75,79], 2010-2019 (n = 36) [33,35,37,39-47,50-54,57,60-64,66,68,69,71,73,74,76-78,80-83], and 2020-2021 (n = 6) [34,48,58,59,65,84]. The funding sources for the studies were - extramural (e.g., academic, government, non-profit, commercial) (n = 28) [36,37,39-44,47,51-53,55,56,59,60,64,66,69-71,75-78,81,83,84], internal (no extramural funding) (n = 3) [34,58,82], and not reported (n = 22) [33,35,38,45,46,48-50,54,57,61-63,65,67,68,72-74,79,80,85].

Study Outcomes

The studies used various outcome measures for MSDs, including validated patient-reported outcomes (e.g., Nordic Musculoskeletal Questionnaire) and administrative measures (e.g., work absenteeism), as well as study-specific measures that have not been validated. Outcomes for HBDs were mainly study-specific and not validated for general use.

Evidence Level and Study Quality

Study level and quality are shown in the Appendices. The evidence level of the uncovered studies was as follows: level 2 (prospective observational cohort) (n = 7) [39,42-44,59,60,84], level 3 (case-control) (n = 6) [41,55,71,76,77,83], level 3 (retrospective cohort) (n = 1) [56], and level 4 (cross-sectional) (n = 39) [33-38,40,45-54,57,58,61-70,72-75,78-82,85]. No level 1 studies were found, which precluded making moderate or strong empirical evidence statements, confirmatory interpretations about causal relationships, and conclusions about the effectiveness of HBD interventions for managing extremity and general MSDs. Study quality for the uncovered studies was as follows: good (n = 8) [39,42-44,56,59,60,84], fair (n = 43) [33-37,40,41,45-48,50-55,57,58,61-83,85], and poor (n = 2) [38,49].

Empirical Evidence Statements

Evidence was uncovered in support of significant relationships between many HBD categories and sub-categories with extremity and general MSDs as detailed in Table 1. For 19 pairwise comparisons, weak evidence supports a relationship indicating that poor HBDs are related to an increased risk of extremity and general MSDs. For example, this review found that poor air quality at work is related to increased risk of upper extremity MSDs [51,57,71,77]. On contrary, for three comparisons, weak evidence indicates that poor HBDs are not related to an increased risk of extremity and general MSDs. For example, this review found that poor lighting at work is not associated with increased risk of lower extremity MSDs [61,62,66]. Conflicting evidence was found for six comparisons. For example, this review found conflicting evidence regarding the relationship between uncomfortably warm temperatures at work or nonspecific locations and increased risk of upper extremity MSDs. Some studies or analyses within studies support this relationship [41,42,46,74], while others do not [40-42,59,74]. No evidence was found for five comparisons.

**Table 1:** Empirical evidence statements for relationship of healthy building determinants (HBDs) with upper extremity, lower extremity, and general musculoskeletal disorders (MSDs). Overall work environment = combination of various HBDs. General MSDs = MSD of unspecified or general body region. Risk for UE, LE, or general MSDs and related outcomes. Evidence statement: yes = supports evidence statement, no = does not support evidence statement. MSDs: musculoskeletal disorders; UE: upper extremity; LE: lower extremity

HBD	Upper extremity MSDs	Lower extremity MSDs	General MSDs
Air quality and ventilation	Poor air quality at work is related to increased risk of UE MSDs. Evidence: weak - yes [51,57,71,77]. No [49,77].	Poor air quality at home or work is related to increased risk of LE MSDs. Evidence: conflicting - yes [64,71]. No [53,66,71].	Poor air quality at work is related to increased risk of general MSDs. Evidence: weak - yes [33,38,69,83]. No [33,38,55].
Dust and pests	Dust complaint or exposure at work is related to increased risk of UE MSDs. Evidence: weak - yes [57,71]. No [77].	Dust exposure at work is related to increased risk of LE MSDs. Evidence: conflicting - yes [71]. No [62,71].	Dust exposure at work is related to increased risk of general MSDs. Evidence: conflicting - yes: [33]. No [33].
Lighting and views	Poor lighting at work is related to increased risk of UE MSDs. Evidence: conflicting - yes [34,45,49,57,58,74]. No [49,61,72,74,77].	Poor lighting at work is NOT related to increased risk of LE MSDs. Evidence: weak - yes [61,62,66]. No [62,66].	Evidence: none.
Moisture	Uncomfortable moisture (dampness, humidity) at work is related to increased risk of UE MSDs. Evidence: weak - yes [46]. No: none.	Uncomfortable moisture (dampness, humidity) at work is related to increased risk of UE MSDs. Evidence: weak - yes [46]. No: none.	Evidence: none.
Noise	Increased noise at work is related to increased risk of UE MSDs. Evidence: weak - yes [45,52,57,74,77]. No [49,72,74].	Increased noise at home or work is related to increased risk of LE MSDs. Evidence: conflicting - yes [52]. No [62,66].	Increased noise at work is related to increased risk of general MSDs. Evidence: weak - yes [35]. No: none.
Safety and security	Poor safety at work is related to increased risk of UE MSDs. Evidence: weak - yes [80]. No: none.	Evidence: none	Poor safety at work is related to increased risk of general MSDs. Evidence: weak - yes [60,82]. No: none.
Thermal Health: Uncomfortable	Uncomfortable temperature at work is related to increased risk of UE MSDs. Evidence: weak - yes [34,57,59,72]. No [37,72,77].	Uncomfortable temperature at work is related to increased risk of LE MSDs. Evidence: weak - yes [37,66]. No [66].	Evidence: none.
Thermal health: cold	Uncomfortably cold temperature at work or nonspecific location is related to increased risk of UE MSDs. Evidence: weak - yes [36,39,46-48,50,59,67,70,75,76,84]. No [36,50,67,70,76].	Uncomfortably cold temperature at work or nonspecific location is related to increased risk of LE MSDs. Evidence: weak - yes [36,46-48,50,67,70,85]. No [47,50,67,70,85].	Uncomfortably cold temperature at work or nonspecific location is related to increased risk of general MSDs. Evidence: weak - yes [68,70,78,81,83]. No: none.
Thermal health: warm	Uncomfortably warm temperature at work or nonspecific location is related to increased risk of UE MSDs. Evidence: conflicting - yes [41,42,46,74]. No [40-42,59,74].	Uncomfortably warm temperature at work or nonspecific location is related to increased risk of LE MSDs. Evidence: weak - yes [46,85]. No [62].	Uncomfortably warm temperature at work is NOT related to increased risk of general MSDs. Evidence: weak - yes [68]. No: none.
Water quality	Drinking poor quality water at nonspecific location is related to increased risk of UE MSDs. Evidence: weak - yes [44]. No: none.	Drinking poor quality water at home or nonspecific location is NOT related to increased risk of LE MSDs. Evidence: weak - yes [43,56,63]. No [43,56].	Drinking poor quality water at work is related to increased risk of general MSDs. Evidence: weak - yes [73]. No: none.
Overall work environment	Poor overall work environment including HBDs is related to increased risk of UE MSDs. Evidence: weak - yes [54,77,79]. No: none.	Evidence: none.	Poor overall work environment including HBDs is related to increased risk of general MSDs. Evidence: Weak - yes [65]. No: none.

For upper extremity MSDs, weak evidence indicates that a positive relationship (i.e., poor HBDs are related to an increased risk of MSDs) exists between nine HBD categories and sub-categories (air quality and ventilation, dust and pests, moisture, noise, safety and security, thermal health (cold, uncomfortable), water quality, overall work environment), and upper extremity MSDs. Conflicting evidence was found for two HBDs (lighting and views, thermal health {warm}). The evidence for lower extremity MSDs was mixed. Weak evidence indicates that a positive relationship exists between four HBD categories and sub-categories (moisture, thermal health {cold, warm, uncomfortable}) and lower extremity MSDs. Weak evidence indicates that an inverse relationship exists between two HBD categories and sub-categories (lighting and views, water quality) and lower extremity MSDs. Conflicting evidence was found for three HBD categories and sub-categories (air quality and ventilation, dust and pests, and noise). No evidence was found for two HBDs (safety and security, overall work environment). For general MSDs, weak evidence indicates that a positive relationship exists between six HBD categories and sub-categories (air quality and ventilation, noise, safety and security, thermal health {cold}, water quality, overall work environment) and general MSDs. Weak evidence indicates that an inverse relationship exists between one HBD category (thermal health {warm}) and general MSDs. Conflicting evidence was found for one HBD category (dust and pests). No evidence was found for three HBD categories and sub-categories (lighting and views, moisture, and thermal health {uncomfortable}).

Discussion

General Interpretation

The current systematic review found 53 studies on the relationship of several HBDs with extremity and general MSDs. More than 60% (32/53) of these studies were published over the last decade and were carried out in diverse countries, settings, and populations, thus the attention given to the HBD-MSD relationship is increasing. This review builds upon our previous work to provide useful starting points about the HBD-MSD relationship. These findings can enhance awareness and help inform future research and public health efforts aimed at addressing suboptimal HBDs through appropriate interventions to improve the lives of those suffering from MSDs [13].

The awareness of the HBD-MSD relationship raised through the current review may also be useful to avoid unintended harm, particularly as the field progresses beyond its early stages. Healthy building initiatives evolved from prior efforts about the relationship between the built environment and human health, which typically have had a positive impact. However, sometimes these efforts had unintended consequences that created human harm. For example, attempts to improve the energy efficiency of buildings in the 1970s resulted in "sick building syndrome" (SBS) and its array of negative health consequences [112]. The SBS example highlights the need to focus on preventing unintended harm when transforming the built environment to optimize the management of MSDs. Learning from the past to sustain present efforts and inform the future is crucial to prevent unintended harm while raising awareness of human health within the indoor built environment.

Evidence from the current systematic review generally indicates that HBDs are related to risk of extremity and general MSDs. That is, poor HBDs are associated with increased risk of MSDs (in other words, as HBDs worsen, the risk of MSDs increases). Overall, the most consistent evidence in support of this statement was found for upper extremity and general MSDs, yet mixed evidence was found for lower extremity MSDs. When comparing the evidence across the various HBDs, consistent evidence supporting a positive relationship with higher risk of extremity and general MSDs was found for thermal health (cold), air quality and ventilation, thermal health (uncomfortable), moisture, safety and security, noise, and overall work environment. However, mixed evidence was found for dust and pests, lighting and views, thermal health (warm), and water quality.

The findings of the current review, combined with our other review examining the HBD relationship with back pain and neck pain, provide a comprehensive initial assessment of the association of numerous HBDs with a wide range of MSDs [13]. When considered together, the cumulative findings of the current review and our other review are largely consistent, particularly for back pain, neck pain, upper extremity MSDs, and general MSDs, as well as the HBDs of air quality and ventilation, moisture, thermal health (cold, uncomfortable), and overall work environment. However, inconsistencies in the evidence are noted for lower extremity MSDs, as well as the HBDs of dust and pests, lighting and views, noise, thermal health (warm), and water quality. When considering the current review and previous review together, the most studies, in terms of number of studies, were uncovered for thermal health, followed by air quality and ventilation, and lighting and views, while the fewest studies were found for safety and security.

The noted differences among the various musculoskeletal regions (e.g., upper extremity compared to lower extremity) and HBD categories and sub-categories (e.g., air quality and ventilation compared to noise) assessed in the current review are challenging to explain and require additional research. Possible explanations for these differences are factors inherent in the populations assessed. For example, most of the studies uncovered in the current review were conducted on workers whose primary job tasks involved relatively more upper extremity use compared to lower extremity. Thus, it is possible that the musculoskeletal regions required for a particular occupation may be most impacted by the built environment and the activity required within that environment.

The level of studies uncovered in this systematic review limits the ability to conduct a full-scale causality assessment using Hill's criteria [113]. Nevertheless, biological plausibility seems reasonable for several of the uncovered HBD-MSD relationships. For example, the current review and our previous review found evidence suggesting that uncomfortably cold indoor temperature was related to an increased risk of MSDs, which was not found for uncomfortably warm indoor temperature [13]. Possible explanations for these findings are that cold impedes muscle and joint movement [114,115], and people suffering from chronic MSDs are hypersensitive to cold [116,117].

Another example of biological plausibility that may help explain the relationship between HBDs and MSDs is the association of environmental tobacco smoke with MSDs, as found in two studies of this review [64,69]. As noted by Pisinger et al., tobacco smoke includes various toxic chemicals and gases, which can negatively impact musculoskeletal tissue perfusion and nutrition, and result in inadequate responses to mechanical stressors [69,118]. Moreover, tobacco smoke causes an increase in inﬂammatory cytokines and attenuation of chondrocyte activity, which may inhibit recovery and growth of musculoskeletal tissues [119].

Another explanation for the uncovered relationships is that HBDs are directly associated with other MSD risk factors that were not accounted for in the reviewed studies. For example, the current review and our previous review found that poor air quality was related to an increased risk of MSDs [13]. In this case, it is plausible that air quality may not be a direct marker for MSDs. Rather, air quality could be a direct marker for respiratory function, which in turn is a direct marker for MSDs. In agreement with this observation, other work suggests that poor indoor air quality contributes to tissue hypoxia [120], and is related to sick-building syndrome [121], which is associated with MSDs, such as muscle pain [121]. Moreover, disordered breathing is associated with aberrant carbon dioxide and oxygen physiology [122], and poorer functional movement quality [123], which in turn are associated with an increased risk for MSDs [122,124]. Similarly, the current review's and previous review's [13] finding that being annoyed with noise from neighbors is associated with an increased risk of extremity MSDs is likely best explained by accounting for other health and environmental aspects that were not measured but may be relevant to residents of multi-story housing units [52].

Limitations

The current systematic review has limitations that are similar to our previous review, which preclude widespread generalizability [13]. The uncovered evidence was mostly from lower-level evidence and no level 1 studies (e.g., controlled trials) were found, thus the impact of interventions targeting the HBD-MSD interface on health outcomes is unknown. Also, no evidence was uncovered for five of the 33 pairwise comparisons for the HBD-MSD relationships, and several comparisons had minimal studies to formulate empirical evidence statements. Furthermore, comparisons among the studies were challenging and meta-analysis was not viable because different outcomes were measured across the studies. Moreover, it is likely that other indoor HBDs exist and may be associated with MSDs, in addition to those assessed in this review, as well as those that may be inherent to the outdoor environment. Also, the studies did not adequately assess residential settings. Finally, the interrelationships of various HBDs and other factors, such as those reflecting what is put into the building rather than the building itself (e.g., ergonomics, wide array of biopsychosocial factors, such as HBDs), that may impact MSD development, recovery, and prognosis were not examined.

Implications for Practice and Policy

While the field examining the HBD-MSD relationship is in its infancy, the findings of this systematic review are useful for future research, development, and public health efforts aimed at attenuating the negative impact of MSDs within the indoor built environment [13]. These findings will help create awareness among various stakeholders involved with enhancing, and who may benefit from, the human-building-environment interface within the HBD-MSD domain, such as companies, employers, employees, property owners, tenants, patients, clinicians, and policymakers. For tenants, patients, and employees, enhancing the HBD-MSD domain could result in improved quality of life, function, and productivity [125]. For employers, these enhancements could improve measures related to job performance and employee retention [125,126]. For property owners, upgrades to human-building-environment interface within the HBD-MSD domain could result in financial gains [125], such as higher rental fees [16,31], enhanced tenant satisfaction and retention [13], and reduced risk of injury or poor health claims against owners and insurance companies. For policymakers, as well as organizations with ecological, social, and governance missions, implementation of practices to optimize the HBD-MSD domain can have wide-ranging impact on reducing human disability related to the built environment [13].

Future Research

Expanding on the current review's findings through future research would be valuable to inform policy and practice, and foster implementation of interventions designed to positively impact the indoor built environment by augmenting HBDs and reducing MSDs [13]. As detailed in a previous review, causality of the HBD-MSD relationships needs to be examined [13]. Interventions targeting HBDs need to be assessed for safety and effectiveness health outcomes for MSD management through level 1 studies, such as randomized controlled trials. Cost-effectiveness and return on investment measures for HBD-related products and services need to be modeled in health economic evaluations. Studies assessing the rising demand for working from home versus working in the office on MSDs would be useful [127]. Moreover, diverse biopsychosocial, demographic, ergonomic, and comorbidity factors should be assessed in terms of their effect on the HBD-MSD relationships.

## Conclusions

Musculoskeletal disorders are a substantial societal burden and various factors affect their causation, recovery, and prognosis, which may include elements that have not been fully researched, such as indoor environmental quality. This review systematically examined the peer-reviewed literature on the relationship of eight HBDs with extremity and general MSDs. The search uncovered 53 eligible studies with 178,532 participants. A total of 74.6% (39/53) of the studies were cross-sectional and 81.1% (43/53) were fair quality. Overall, the majority of uncovered evidence indicates that HBDs are related to risk of extremity and general MSDs. Nineteen comparisons support that as HBDs worsen, the risk of MSDs increases. Six comparisons had conflicting evidence. Three comparisons support that poor HBDs are not related to increased risk of extremity and general MSDs. Five comparisons had no evidence. This systematic review builds upon previous work to provide useful starting points to enhance awareness about the HBD-MSD relationship. These findings can help inform research and public health efforts aimed at addressing suboptimal HBDs through appropriate interventions to improve the lives of those suffering from MSDs.

## Appendices

**Table 2:** PubMed search strategy.

Search number	Search details
47	19 AND 25 AND 43 (filters: abstract, humans, English)
43	26 OR 27 OR 28 OR 29 OR 30 OR 31 OR 32 OR 33 OR 34 OR 35 OR 36 OR 37 OR 38 OR 39 OR 40 OR 41 OR 42
42	"toes"(MeSH Terms) OR "toes"(All Fields) OR "toe"(All Fields) OR "toe joint"(MeSH Terms) OR ("toe"{All Fields} AND "joint"{All Fields}) OR "toe joint"(All Fields)
41	"fingers"(MeSH Terms) OR "fingers"(All Fields) OR "finger"(All Fields) OR "finger joint"(MeSH Terms) OR ("finger"{All Fields} AND "joint"{All Fields}) OR "finger joint"(All Fields)
40	"hand"(MeSH Terms) OR "hand"(All Fields) OR "hands"(All Fields) OR "hand joints"(MeSH Terms) OR ("hand"{All Fields} AND "joints"{All Fields}) OR "hand joints"(All Fields) OR ("hand"{All Fields} AND "joint"{All Fields}) OR "hand joint"(All Fields)
39	"wrist"(MeSH Terms) OR "wrist"(All Fields) OR "wrists"(All Fields) OR "wrist joint"(MeSH Terms) OR ("wrist"{All Fields} AND "joint"{All Fields}) OR "wrist joint"(All Fields)
38	"forearm"(MeSH Terms) OR "forearm"(All Fields) OR "forearms"(All Fields)
37	"elbow"(MeSH Terms) OR "elbow"(All Fields) OR "elbows"(All Fields) OR "elbow joint"(MeSH Terms) OR ("elbow"{All Fields} AND "joint"{All Fields}) OR "elbow joint"(All Fields)
36	"arm"(MeSH Terms) OR "arm"(All Fields) OR "arms"(All Fields)
35	"shoulder"(MeSH Terms) OR "shoulder"(All Fields) OR "shoulders"(All Fields) OR "shoulder joint"(MeSH Terms) OR ("shoulder"{All Fields} AND "joint"{All Fields}) OR "shoulder joint"(All Fields)
33	"foot"(MeSH Terms) OR "foot"(All Fields) OR "feet"(All Fields) OR "foot joints"(MeSH Terms) OR ("foot"{All Fields} AND "joints"{All Fields}) OR "foot joints"(All Fields) OR ("foot"{All Fields} AND "joint"{All Fields}) OR "foot joint"(All Fields)
32	"ankle"(MeSH Terms) OR "ankle"(All Fields) OR "ankles"(All Fields) OR "ankle joint"(MeSH Terms) OR ("ankle"{All Fields} AND "joint"{All Fields}) OR "ankle joint"(All Fields)
31	"leg"(MeSH Terms) OR "leg"(All Fields) OR "legs"(All Fields)
30	"knee"(MeSH Terms) OR "knee"(All Fields) OR "knees"(All Fields) OR "knee joint"(MeSH Terms) OR ("knee"(All Fields) AND "joint"(All Fields)) OR "knee joint"(All Fields)
29	"thigh"(MeSH Terms) OR "thigh"(All Fields) OR "thighs"(All Fields)
28	"hip"(MeSH Terms) OR "hip"(All Fields) OR "hips"(All Fields) OR "hip joint"(MeSH Terms) OR ("hip"{All Fields} AND "joint"{All Fields}) OR "hip joint"(All Fields)
27	"limbs"(All Fields) OR "limb"(All Fields) OR "limbed"(All Fields)
26	"extremities"(MeSH Terms) OR "extremities"(All Fields) OR "extremity"(All Fields)
25	20 OR 21 OR 22 OR 23 OR 24
24	("musculoskeletal"(All Fields) AND "conditions"(All Fields)) OR "musculoskeletal conditions"(All Fields) OR ("musculoskeletal"{All Fields} AND "condition"{All Fields}) OR "musculoskeletal condition"(All Fields)
23	("musculoskeletal"{All Fields} AND "disorders"{All Fields}) OR "musculoskeletal disorders"(All Fields) OR ("musculoskeletal"{All Fields} AND "disorder"{All Fields}) OR "musculoskeletal disorder"(All Fields)
22	"musculoskeletal pain"(MeSH Terms) OR ("musculoskeletal"{All Fields} AND "pain"{All Fields}) OR "musculoskeletal pain"(All Fields) OR ("musculoskeletal"{All Fields} AND "pains"{All Fields}) OR "musculoskeletal pains"(All Fields)
21	"musculoskeletal diseases"(MeSH Terms) OR ("musculoskeletal"{All Fields} AND "diseases"{All Fields}) OR "musculoskeletal diseases"(All Fields) OR ("musculoskeletal"{All Fields} AND "disease"{All Fields}) OR "musculoskeletal disease"(All Fields)
20	"musculoskeletal system"(MeSH Terms) OR ("musculoskeletal"{All Fields} AND "system"{All Fields}) OR "musculoskeletal system"(All Fields) OR ("musculoskeletal"{All Fields} AND "systems"{All Fields}) OR "musculoskeletal systems"(All Fields)
19	1 OR 2 OR 3 OR 4 OR 5 OR 6 OR 7 OR 8 OR 9 OR 10 OR 11 OR 12 OR 13 OR 14 OR 15 OR 16 OR 17 OR 18
18	"safety"(MeSH Terms) OR "safety"(All Fields) OR "safeties"(All Fields)
17	"security"(All Fields) OR "securities"(All Fields)
16	"temperature"(MeSH Terms) OR "temperature"(All Fields)
15	"thermal"(All Fields) OR "thermal health"(All Fields)
14	"moisture"(All Fields) OR "moistures"(All Fields)
13	"pest"(All Fields) OR "pests"(All Fields)
12	"dust"(MeSH Terms) OR "dust"(All Fields) OR "dusts"(All Fields)
11	"water quality"(MeSH Terms) OR "water quality"(All Fields)
10	"noise"(MeSH Terms) OR "noise"(All Fields) OR "noises"(All Fields)
9	"lighting"(MeSH Terms) OR "lighting"(All Fields) OR "lightings"(All Fields)
8	"ventilation"(MeSH Terms) OR "ventilation"(All Fields) OR "ventilations"(All Fields) OR "ventilate"(All Fields)
7	"tobacco smoke pollution"(MeSH Terms) OR "tobacco smoke pollution"(All Fields) OR "second hand smoke"(All Fields) OR "environmental tobacco smoke"(All Fields)
6	"air pollution"(MeSH Terms) OR "air pollution"(All Fields) OR "air quality"(All Fields)
5	"built environment"(MeSH Terms) OR "built environment"(All Fields)
4	"environmental illness"(MeSH Terms) OR "environmental illness"(All Fields) OR "environmental illnesses"(All Fields)
3	"indoor environmental quality"(All Fields) OR "indoor environment"(All Fields)
2	"sick building syndrome"(MeSH Terms) OR "sick building syndrome"(All Fields)
1	"healthy buildings"(All Fields) OR "healthy building"(All Fields)

**Table 3:** Characteristics and outcomes of included studies. P-value > 0.05 is not significant. General=MSD of unspecified or general body region. ANOVA: analysis of variance; BMI: body mass index; CI: confidence interval; ETS: environmental tobacco smoke; F: female; GI: gastrointestinal; HBD: healthy building determinant; HCP: health care provider; ICD: international classification of disease; IQR: interquartile range; LE: lower extremity; M: male; MSD: musculoskeletal disorder; MSK: musculoskeletal; NIOSH: US National Institute for Occupational Safety and Health; NMQ: Nordic Musculoskeletal Questionnaire (original or adapted); NR: not reported; NRS: numerical rating scale; OMPQ: Orebro Musculoskeletal Pain Questionnaire; OR: odds ratio; OWE: overall work environment; PR: prevalence ratio; PRO: patient-reported outcome; RR: relative risk; UE: upper extremity; VAS: visual analog scale

Author, year, country, funding source	Population studied, sample size (gender), age	Inclusion/exclusion criteria	HBD type, outcome assessed	MSD type, outcome assessed	Analysis procedures, outcomes
Ahmad et al. in 2014 [33], Bangladesh, NR	Lead acid battery workers, 118 (NR), 31.3 ± 11.4 years.	Inclusion: worker at one of 15 lead acid battery industries in Dhaka city. Employed ≥ 1 year. Provided informed consent. Exclusion: NR.	Air quality and ventilation, dusts, and pests. Serum blood lead level (mg/dL).	General. Prevalence of limb numbness and limb pain: yes/no (PRO).	T-test, workers with limb numbness had higher blood lead levels than those without limb numbness (blood lead level - yes: 73.47 ± 34.32 mg, no: 62.32 ± 22.73 mg, p = 0.044). No significant difference in blood lead levels between workers with and without limb pain.
Alhusuny et al. in 2021 [34], Australia and New Zealand, No extramural funding	Surgeons, 290 (138 F, 152 M), 46.2 ± 10.9 years.	Inclusion: surgeon of any age, sex, or surgical title, who worked as main or assistant surgeon in operating theatre performing 2D and/or 3D laparoscopic surgery and/or robotic surgery. Exclusion: NR.	Lighting and views, noise, thermal health. Sensitivity to light: 5-point Likert scale (PRO); frequency of adjusting lighting, ambient temperature, ambient noise at work: 5-point Likert scale (PRO).	UE. Neck-shoulder pain in past 12 months: NMQ (PRO).	Logistic regression, increased sensitivity to light was significantly associated with increased risk of neck-shoulder pain in past 12 months (OR 3.2, 95% CI 1.7-5.8, p < 0.001). Frequent action to adjust temperature in room was significantly associated with increased risk of neck-shoulder pain in past 12 months (OR 2.6, 95% CI 1.1-5.9, p = 0.024). Noise: NR.
Assunção and Abreu in 2017 [35], Brazil, NR	General population, 60,202 (33,171 F, 27,031 M), 35-44 year (median).	Inclusion: residents of private permanent households in Brazil who completed the National Survey on Health. Age ≥ 18 years. Exclusion: NR.	Noise. Exposure to noise at work: yes/no (PRO).	General. Occurrence of self-reported work-related MSD via initial question "has some doctor ever diagnosed you with WMSD (work-related MSD)?" (PRO).	Logistic regression, exposure to noise at work was significantly associated with increased risk of work-related MSDs (OR 2.16, 95% CI 1.68-2.77, p < 0.001).
Bang et al. in 2005 [36], Norway, the Confederation of Norwegian Business and Industry	Seafood industry workers, 1,767 (760 F, 1,007 M), 39 years (median).	Inclusion: workers from one of 17 seafood industry plants in Norway. Exclusion: NR.	Thermal Health. Feeling of cold at work often: yes/no (PRO).	UE, LE. MSK pain in past 12 months via the question: "have you, during the last 12 months, felt pain from the (neck/shoulders, elbow, wrists/hands, legs)? yes/no (PRO).	Chi-square, feeling cold often at work was associated with an increased risk of pain in the neck/shoulders (felt cold often 81.7%, never felt cold 46.9%, p < 0.001), wrist/hands (felt cold often 55.9%%, never felt cold 23.0%, p < 0.001), and legs (felt cold often 53.8%, never felt cold 26.5%, p < 0.001). No significant difference in risk of elbow pain between those feeling cold at work often and never.
Barro et al. in 2015 [37], Brazil, National Council for Scientific and Technological Development	Poultry processing plant workers, 1,103 (725 F, 378 M), 31-40 years (median).	Inclusion: employees of poultry processing plant located in Southern Brazil. Employed for ≥ 12 months. Working a fixed-shift schedule. Exclusion: been away from work (regardless of reason) for > 10 days. Pregnant.	Thermal health. Work in extreme temperature environment (hot or cold) or moderate thermal environment: administrative data.	UE, LE. MSK pain in past 12 months: NMQ (PRO).	Logistic regression, prevalence ratio (PR). Working in an extreme temperature environment was significant with increased risk of LE pain in females (PR 1.75, 95% CI 1.12-2.71, p < 0.05) and males (PR 2.17, 95% CI 1.12-4.22, p < 0.05). No significant relationship between working in an extreme temperature environment and UE pain.
Carnow and Conibear in 1981 [38], Canada, NR	Smelting factory workers, 1,242 (gender: NR), 31-40 years (median).	Inclusion: hourly employees of smelting factory. Exclusion: on disability leave, worked at smelting factory for ≤ 3 months.	Air quality and ventilation. Fluoride exposure in ambient air at work: exposure risk index (low, medium, high).	General. Prevalence MSK pain disorder history while at current job: yes/no (PRO); current MSK pain frequency: 0-15, categorized as low or high frequency (PRO).	Chi-square. Medium and high exposure to ambient air fluoride (compared to low exposure) was significantly associated with increased prevalence of history of MSK pain disorders (medium exposure: chi-square 37.43, p < 0.001; high exposure: chi-square 42.9, p < 0.001). Medium exposure to ambient air fluoride (compared to low exposure) was significantly associated with increased frequency of current MSK pain (chi-square 14.92, p < 0.001). No significant relationship between high exposure to ambient air fluoride (compared to low exposure) and frequency of current MSK pain.
Coombes et al. in 2015 [39], Australia, National Health and Medical Research Council	Individuals from general population with lateral epicondylitis, 41 (17 F, 28 M), 49.9 ± 7.4 years.	Inclusion: participants enrolled in a randomized controlled trial, unilateral lateral elbow pain of > 30/100 on VAS, >6 weeks duration, aggravated by ≥2 of the following: gripping, palpation, resisted wrist or middle finger extension. Exclusion: injections in past 6 months, physical therapy in past 3 months, concurrent clinical neck or arm pain, radicular or neurological symptoms, systemic arthritis, pregnancy, breast-feeding, contraindication to injection.	Thermal health. Cold pain threshold using thermotest system.	UE. Lateral elbow pain and disability, patient-rated tennis elbow evaluation (PRTEE) (PRO).	Linear regression. Poorer cold pain threshold was significantly associated with higher disability and pain at 2-month follow-up (β 0.77, 95% CI 0.21-1.33, p = 0.008) and 12-month follow-up (β 0.61, 95% CI 0.05-1.17, p = 0.034).
Coronado et al. in 2011 [40], United States, University of Florida Research Opportunity Seed Fund	Individuals from general population seeking surgery for shoulder pain, 59 (24 F, 35 M), 50.4 ± 14.9 years.	Inclusion: ages 18-85 years, shoulder pain, rotator cuff tendinopathy (small, medium, large), adhesive capsulitis, or labral lesion, schedule for arthroscopic shoulder surgery. Exclusion: current neck, elbow, hand, low back, hip knee, or ankle pain, massive or complete rotator cuff tear, shoulder surgery in past year or shoulder pain from previous surgery, shoulder fracture, tumor, infection, chronic or systemic pain disorder, current psychiatric disorder, current GI or renal illness.	Thermal health. Thermal pain sensitivity (threshold and tolerance) using contact thermode and computer-controlled neurosensory analyzer. Comparison within subject, side-to-side.	UE. Brief pain inventory for shoulder pain: 11-point NRS (PRO).	ANOVA, Chi-square. No significant relationships between thermal pain sensitivity and shoulder pain comparing those involved with uninvolved sides.
Coronado et al. in 2014 [41], United States, National Institutes of Health	Individuals from general population with and without shoulder pain, 114 (33 F, 81 M), 30.5 ± 10.0 years.	Inclusion: ages 18-85 years, shoulder pain, rotator cuff tendinopathy (small, medium, large), adhesive capsulitis, or labral lesion, schedule for arthroscopic shoulder surgery. Exclusion: current neck, elbow, hand, low back, hip knee, or ankle pain of > 3 months duration, neurological disorder, history of shoulder osteoarthritis or rheumatoid arthritis, shoulder fracture, tumor, infection, chronic or systemic pain disorder, current psychiatric disorder, current GI or renal illness, shoulder surgery in past year or shoulder pain from previous surgery. Cases were matched by age and sex to healthy controls.	Thermal health. Thermal pain sensitivity (threshold, tolerance, suprathreshold heat pain response (SHPR) using contact thermode, computer-controlled neurosensory analyzer, contact heat evoked potential stimulator. Comparison between subjects, with and without shoulder pain.	UE. Brief pain inventory for shoulder pain: 11-point NRS (PRO).	T-test, rank Sums, and Wilcoxon signed-rank test. SHPR was significantly worse in participants with shoulder pain compared to asymptomatic controls (shoulder pain affected side: 38.3 ± 24.4, shoulder pain non-affected side: 35.0 ± 25.3, asymptomatic controls: 25.0 ± 25.4, p < 0.015). No significant difference in SHPR between affected and non-affected sides in participants with shoulder pain. No significant differences in thermal pain threshold or thermal pain tolerance among participants with shoulder pain (affected and non-affected sides) and asymptomatic controls.
Coronado et al. in 2014 [42], United States, National Institutes of Health	General population, 143 (85 F, 58 M), 23.7 ± 6.7 years.	Inclusion: ages 18-85 years, not performing resistance training for upper extremities for past 6 weeks. Exclusion: current neck or shoulder pain, neurological impairments of UE, current pain medication, history of shoulder surgery.	Thermal health. Thermal pain sensitivity (suprathreshold heat pain Response (SHPR)) using contact heat evoked potential stimulator. Comparison within subject, pre and post exercise-induced pain.	UE. Shoulder pain intensity at rest and activity (dynamic motion, isometric): 0-100 VAS (PRO).	Linear regression, poorer pre-injury SHPR was significantly associated with worse shoulder pain during dynamic motion and isometric activities at 48-hours and 96-hours post-injury (dynamic motion 48-hours: R 0.251, p < 0.05; dynamic motion 96-hours: R 0.437, p < 0.05; isometric 48 hours: R 0.222p < 0.05; isometric 96 hours: R 0.320, p < 0.05). No significant relationships between SHPR and rest related shoulder pain.
Dahl et al. in 2014 [43], Norway, Research Council of Norway	General population, 18,130 (9,242 F, 8,888 M), 50.2 ± 15.8 years.	Inclusion: ages 50-85 y during 1994-2000, treated for hip fracture in Norwegian hospitals, data available in Norwegian Epidemiologic Osteoporosis Studies database, completed information on cadmium, lead, and aluminum in their drinking water. Exclusion: NR.	Water quality. Average concentration of heavy metals (aluminum, cadmium, lead) in drinking water: categorized as dichotomous variable.	LE. Hospital records for hip fracture based on ICD diagnosis codes for hip fracture.	Poisson regression, higher concentrations of heavy metals in drinking water were significantly associated with higher rates of hip fractures in males (aluminum: IRR 1.08, 95% CI 1.02-1.15, p ≤ 0.05; cadmium: IRR 1.09, 95% CI 1.00-1.19, p ≤ 0.05; lead: IRR 1.08, 95% CI 1.00-1.17. No significant relationships between concentrations of heavy metals in drinking water and hip fracture in females.
Dahl et al. in 2013 [44], Norway, Research Council of Norway	General population, 19,067 (13,629 F, 5,438 M), 50-85 years.	Inclusion: ages ≥ 20 years, participant in Cohort of Norway (CONOR) between 1994-2003, forearm fracture identified in CONOR database. Exclusion: NR.	Water quality. average pH of municipal drinking water.	UE. Response to question: "have you ever broken (fractured) your wrist/forearm?": yes/no (PRO).	Logistic regression, consuming municipal drinking water with pH < 7.0 compared to pH ≥ 7.0 was significantly associated with increased risk of wrist fracture (female: OR 1.14, 95% CI 1.08-1.19, p < 0.05; male: OR 1.19, 95% CI 1.14-1.25, p < 0.05).
d'Errico et al. 2010 [45], Italy, NR	Call center workers, 775 (560 F, 195 M), 30-39 years (median).	Inclusion: call center workers from Turin region of Italy. Exclusion: NR.	Lighting and views, noise. Orege questionnaire and job content questionnaire to assess workplace desk lighting and noise (PRO).	UE. UE MSK symptoms during past 28 days in which HCP was consulted or medications were taken (PRO).	Poisson regression, RR. Inadequate desk lighting was significantly associated with increased risk of symptoms in the neck-shoulder (RR 1.47, 95% CI 1.25-1.73, p < 0.05) and elbow-wrist/hand (RR 1.76, 95% CI 1.18-2.61, p < 0.05). Continuously elevated noise was significantly associated with increased risk of symptoms in the neck-shoulder (RR 1.50, 95% CI 1.27-1.77, p < 0.05) and elbow-wrist/hand (RR 1.98, 95% CI 1.33-2.93, p < 0.05).
Douphrate et al. in 2016 [46], United States, NR	Dairy parlor workers (milkers), 450 (48 F, 402 M), 30.3 ± 9.0 years.	Inclusion: ages ≥ 20 years, dairy farm workers (milkers) from 32 large-herd dairy farms in 5 westerns states in US. Exclusion: NR.	Moisture, thermal health. Job questionnaire including items on hot, humid, cold, wet conditions: 0-10 scale (PRO).	UE, LE. MSK symptoms in past 12 months: NMQ (PRO).	Poisson regression. Hot, cold, humid, wet work environments were significantly associated with MSK symptoms in the upper extremities (PR 1.48, 95% CI 1.10-1.98, p < 0.05) and lower extremities (PR 1.56, 95% CI 1.05-2.32, p < 0.05).
Farbu et al. in 2019 [47], Norway, UiT - The Arctic University of Norway	General population, 6,533 (3,321 F, 3,212 M), 30-67 years.	Inclusion: residents from municipality of Tromso in Norway. Exclusion: retired, above retirement age (age 67 years), on full-time disability benefits, missing values in survey.	Thermal health. Work in cold environment at least 25% of time: yes/no (PRO).	UE, LE. Persistent or recurring pain in UE and LE in past 3 months: yes/no (PRO).	Logistic regression, work in cold environment ≥ 25% of time was significantly associated with increased risk of persistent or recurring pain in the shoulder (OR 1.96, 95% CI 1.58-2.42, p < 0.05), arm (OR 1.93, 95% CI 1.49-2.50, p < 0.05), hand (OR 1.66, 95% CI 1.19-2.32, p < 0.05), hip (OR 1.59, 95% CI 1.19-2.12, p < 0.05), and leg (OR 1.87, 95% CI 1.47-2.40, p < 0.05). No significant relationship between work in cold environment ≥ 25% of time and foot pain.
Ghani et al. in 2020 [48], Pakistan, NR	Cold storage facility workers, 200 (0 F, 200 M).	Inclusion: workers in frozen food cold storage facilities in Lahore, India, age > 18 years, comprised of those exposed to cold indoor work environment (n = 100) and those not exposed to cold indoor work environment (n = 100). Exclusion: NR.	Thermal health. Work in cold environment: yes (exposed group, workplace temperature = -20 to - 30°C), No (control group, workplace temperature = NR).	UE, LE. MSK symptoms in past 12 months: NMQ (PRO).	ANOVA, RR. Work in cold environment was significantly associated with increased risk of pain in the shoulders (RR 151.00, 95% CI 9.48-2403.28, p = 0.0004), elbows (RR 10.40, 95% CI 4.33-2434.82, p = 0.0001), wrist/hands (RR 23.33, 95% CI 7.59-71.64, p = 0.0001), hips/thighs (RR 111.00, 95% CI 6.95-1772.51, p = 0.001), knees (RR 6.87, 95% CI 3.45-13.67, p = 0.001), and ankles/feet (RR 3.53, 95% CI 2.13-5.83, p = 0.001).
Ignatius et al. in 1993 [49], China, NR.	Typist workers, 170 (170 F, 0 M), 31.5 ± 7.0 years.	Inclusion: typists working at government housing department. Exclusion: NR.	Air quality and ventilation, lighting and views, noise. Poor lighting, noisy environment, polluted air: yes/no/unsure interview (PRO).	UE. MSK symptoms and fatigue - point prevalence: interview (PRO).	Chi-square, T-test, Logistic regression. Poor lighting was significantly associated with increased risk of hand-finger pain (OR 2.5, p = 0.016). No significant relationship between poor lighting and arm-elbow pain. No significant relationship between noisy environment or polluted air and hand-finger pain or arm-elbow pain.
Inaba et al. in 2011 [50], Japan, NR	Sorting goods workers, 133 (133 F, 0 M), 25-68 years.	Inclusion: sorting goods workers at 2 companies in Japan, comprised of cold storage goods sorting workers (n = 47) and dry goods sorting workers (n = 86). Exclusion: NR.	Thermal health. Work in cold environment: yes (exposed group, cold storage goods sorting, surface temperature = -3 to -9°C; ambient temperature: 22-23°C), No (control group, dry goods sorting, surface temperature = 27°C; ambient temperature: 25-26°C).	UE, LE. MSK symptoms prevalence during prior summer months: questionnaire (PRO).	Chi-square, T-test, ANOVA. Compared to non-exposed workers, cold-exposed workers had significantly greater prevalence of pain in the wrist (exposed 68%, non-exposed 41%, p < 0.01), elbow (exposed 53%, non-exposed 35%, p < 0.05), and foot (exposed 64%, non-exposed 44%, p < 0.05). No significant difference in prevalence of pain in the finger, arm, shoulder, and knee between cold-exposed workers and non-exposed workers.
Janwantanakul et al. in 2010 [51], Thailand, Social Security Ofﬁce of Thailand	Office workers, 1,185 (807 F, 378 M), 35.2 ± 8.4 years.	Inclusion: office workers from 54 workplaces in Bangkok, Thailand, ≥ 1 year in current position. Exclusion: NR.	Air quality and ventilation, dusts, and pest, lighting and views, thermal health. Questionnaire including items on work environment conditions (temperature, light intensity, noise level, air circulation): agree/disagree (PRO).	UE. MSK symptoms in past 12 months: NMQ (PRO).	Chi-square, logistic regression. Disagreeing with the statement that "air circulation in the office is good" was significantly associated with increased risk of experiencing MSK symptoms in the wrist/hand (OR 1.43, 95% CI 1.06-1.94, p = 0.21). Relationships of temperature, light intensity, and noise level with wrist/hand MSK: NR. Relationships of temperature, light intensity, noise level, and air circulation with shoulder and elbow MSK: NR.
Jensen et al. in 2019 [52], Denmark, Realdania, Danish National Building Fund, Aase and Ejnar Danielsen’s Fund	General population, 3,509 (1,985 F, 1,524 M), 25-44 years (median).	Inclusion: ages ≥ 16 years, listed in Danish Civil Registration System, data available from Danish Health and Morbidity Survey in 2017. Exclusion: NR.	Noise. Questionnaire inquiring about noise annoyance: very annoyed/slightly annoyed/no (PRO).	UE, LE. Questionnaire inquiring about bothersome extremity pain or discomfort in past 2: very bothered/slightly bothered/no (PRO).	Logistic regression. Being very annoyed or slightly annoyed with neighbor noise was significantly associated with an increased risk for pain or discomfort in the shoulder or neck (very annoyed: 1.73, OR 1.22-2.45, 95% CI, p = 0.0016; slightly annoyed: OR 1.32, 95% CI 1.06-1.65, p = 0.0016) and arms, hands, legs, knees, hips, or joints (very annoyed: 2.23 OR, 95% CI 1.57-3.17, p < 0.0001; slightly annoyed: OR 1.29, 95% CI 1.03-1.61, p < 0.0001).
Kang et al. in 2016 [53], South Korea, Jaseng Medical Foundation	Individuals from general population with osteoarthritis, 9,042 (5,136 F, 3,906 M), ≥ 50 years.	Inclusion: ages ≥ 50 years, data available from 5th Korean National Health and Nutrition Examination Survey in 2010-2012, received knee or hip radiographs, completed survey on osteoarthritis and smoking. Exclusion: NR.	Air quality and ventilation. ETS exposure defined by question - "how much are you exposed to indirect smoking at home or at work?" and subsequent questioning (PRO).	LE. Hip or knee osteoarthritis defined by question - "have you experienced knee pain/hip pain for 30 days or longer over the past 3 months?": yes/no (PRO) and radiographic evidence of knee or hip joint osteoarthritis defined as Kellgren-Lawrence grade ≥ 2.	ANOVA, chi-square, logistic regression. No significant relationships between indirect smoking (ETS) and hip or knee osteoarthritis.
Kaufman-Cohen and Ratzon in 2011 [54], Israel, NR	Classical musicians, 59 (30 F, 29 M), 42.9 ± 11.43 years.	Inclusion: classical musicians. Exclusion: NR.	Air quality and ventilation, lighting and views, moisture, noise, thermal health, OWE. NIOSH Generic Job Stress Questionnaire including item on perceived physical environment (sound intensity, ambient temperature, local humidity, ventilation, and illumination) (PRO).	UE. MSK pain in past 12 months: NMQ (PRO).	Linear regression. Perceived physical environment was significantly associated with UE MSDs (R = 0.39, p < 0.01).
Kesavachandran et al. in 2009 [55], India, Uttar Pradesh Council of Science and Technology	Pesticide retail shop workers, 38 (0 F, 38 M), 33.1 ± 8.4 years.	Inclusion: workers in 20 pesticide retail shops in Barabank and Lucknow, India. Exclusion: chronic disease, e.g., tuberculosis, diabetes, thyroid disorder, malignancy. Controls (non-exposed admin workers) were matched to cases on socioeconomic status.	Air quality and ventilation. Assessment of various pesticides including organophosphates, organochlorines, carbamates, and pyrethroids.	General. MSDs identified by medical history and clinical examination.	Chi-square, T-test. No significant relationship between exposure to pesticides and MSDs.
Kurttio et al. in 1999 [56], Finland, Academy of Finland - Research Council of Health, University of Kuopio, Finland Ministry of Social Affairs and Health	General population, 4,449 (3,200 F, 1,249 M), 70-74 years (median).	Inclusion: data available from Population Census of Statistics Finland, born in 1900-1930, lived at same address from 1967-1980, from villages in which > 90% of population were not provided with municipal drinking water system. Exclusion: NR.	Water quality. Quantitative measure (potentiometer) of fluoride concentration from drinking wells.	LE. Hospital discharge registry data on hip fracture based on ICD diagnosis codes for hip fracture.	Cox regression. Exposure to high fluoride concentration in drinking water was significantly associated with increased risk of hip fractures in females ages 50-65 years (RR 2.09, 95% CI 1.16-3.76, p < 0.05). No significant relationship between exposure to fluoride concentration in drinking water in females ages 66-80 years or males ages 50-65 years and 66-80 years and hip fractures.
Magnavita et al. in 2011 [57], Italy, NR	Hospital workers, 1,744 (977 F, 767 M), 44.9 ± 8.9 years.	Inclusion: workers from 3 hospitals in Lazio region of Italy. Exclusion: NR.	Air quality and ventilation, dusts, and pests, lighting and views, noise, Thermal health, OWE. IAQ/MM-040 questionnaire with item on "have you been annoyed in the last 3 months by any of these factors in the workplace?" Categorized for temperature complaints (draughts, too high temperature, too low temperature, temperature changes), noise and light complaints, other environmental complaints (stuffy air, dry air, unpleasant smells, static electricity, passive smoke, dust): no, Sometimes, Often every week (PRO).	UE. MSK pain in past 12 months: NMQ (PRO).	Logistic regression. Environmental complaints were significantly associated with increased risk of UE MSDs (temperature complaints: OR 2.45, 95% CI 1.97-3.03, p < 0.05; noise and light complaints: OR 2.10, 95% CI 1.74-2.55, p < 0.05; other environmental complaints (air, dust): OR 2.85, 95% CI 2.23-3.64, p < 0.05).
Mekonnen et al. in 2020 [58], Ethiopia, no extramural funding	Tailor workers, 419 (27 F, 392 M), 29.2 ± 1.5 years.	Inclusion: self-employed tailors in Gondar Ethiopia, worked for ≥ 12 months prior to enrollment. Exclusion: history of injury, accidents, pregnant.	Lighting and views. Adequacy of light at work dichotomous variable (yes/no) (PRO).	UE. Neck-shoulder pain severity and disability assessed by 7-item questionnaire (PRO).	Logistic regression. Inadequate workplace lighting was significantly associated with increased risk of neck-shoulder pain (OR 5.02, 95% CI 3.50-9.03, p < 0.05).
Miettinen et al. in 2021 [59], Finland, Northern Finland Birth Cohort 1966	Various workers, 6,325 (3,066 F, 3,259 M), ≥ 31 years.	Inclusion: data available from Northern Finland Birth Cohort of 1966, born in Oulu and Lapland provinces of Finland, completed postal questionnaire on work-related factors. Working ≥ 3 days/week at study baseline in 1997. Exclusion: BMI < 18.5, diagnosed with ulnar nerve entrapment or carpal tunnel syndrome at baseline.	Thermal health. Questionnaire inquiring about "Are you exposed to the following in your work environment?" includes factors of heat, cold, temperature changes: yes/no (PRO).	UE. Hospitalization for ulnar nerve entrapment identified from Register for Healthcare, ICD diagnosis codes for ulnar nerve entrapment.	Cox regression. Exposures to cold and temperature changes were significantly associated with increased risk of hospitalization for ulnar nerve entrapment (cold: OR 1.96, 95% CI 1.19-3.49, p < 0.05; temperature changes: OR 2.40, 95% CI 1.47-3.92, p < 0.05). No significant relationship between exposure to heat and hospitalization for ulnar nerve entrapment.
Miranda et al. in 2011 [60], United States, National Institute for Occupational Safety and Health	Nursing home workers, 344 (314 F, 30 M), ≥ 40 years (median).	Inclusion: permanent full- and part-time clinical employees in 12 nursing homes of one company in Maryland and Maine, US, participant in the research initiative - promoting mental and physical health of caregivers through transdisciplinary intervention, within the Center for the Promotion of Health in the New England Workplace. Exclusion: temporary employees, office, laundry, food service, janitorial staff.	Safety and security. Physical assaults at work assessed with question (baseline, 12-, 24-month follow-up): “in the past 3 months, have you been hit, kicked, grabbed, shoved, pushed or scratched by a patient, patient’s visitor or family member while you were at work?” Categorized based on number of events (PRO).	General. MSK symptoms in past 3 months assessed by the item for each body region: experienced pain or aching during the preceding 3 months in lower back, shoulders, wrists or hands, knees: yes/no (PRO). Pain intensity and pain interference with work assessed with 5-point Likert scale (PRO).	Log-binomial regression. At 12-month follow-up, baseline exposure to workplace violence of ≥ 3 physical assaults was significantly associated with increased risk of MSK pain in any body region (PR 1.4, 95% CI 1.2-1.8, p < 0.05), widespread MSK pain in ≥ 3 body regions (PR 2.4, 95% CI 1.3-4.4, p < 0.05), moderate-extreme MSK pain (PR 1.6, 95% CI 1.2-2.1, p < 0.05), and pain interfering with work (PR 1.6, 95% CI 1.1-2.3, p < 0.05). At 24-month follow-up, long-term persistent exposure to workplace violence was significantly associated with increased risk of MSK pain in any body region (PR 1.3, 95% CI 1.0-1.8, p < 0.05), moderate-extreme MSK pain (PR 1.7, 95% CI 1.2-2.4, p < 0.05), and pain interfering with work (PR 2.0, 95% CI 1.3-3.1, p < 0.05). No significant relationship between long-term persistent exposure to workplace violence and widespread MSK pain in ≥ 3 body regions.
Nag et al. in 2015 [61], India, NR	Tobacco factory workers, 450 (300 F, 150 M), 37.5 ± 11.6 years.	Inclusion: tobacco factory workers. Exclusion: NR.	Lighting and views. Work environment, including workspace illumination, assessed with a questionnaire: 5-point Likert scale (PRO).	UE, LE. Prevalence of MSDs assessed with NIOSH checklist (PRO). Pain severity assessed with a questionnaire: 4-point Likert scale (PRO).	Pearson's correlation. No significant relationships between workspace illumination and MSDs of the shoulder, knee, or calf.
Nag et al. in 2010 [62], India, NR	Weaving industry workers, 516 (263 F, 253 M), 39.6 ± 10.4 years.	Inclusion: handloom and power loom weaver workers in Ahmeabad region of India. Exclusion: NR.	Dusts and pests, lighting and views, noise, thermal health.	LE. Prevalence of MSDs assessed with NIOSH checklist (PRO). Pain severity assessed with a questionnaire: 4-point Likert scale (PRO).	Pearson's correlation, logistic regression. Less illumination at work was significantly associated with increased prevalence of MSDs of the knee in female workers (p < 0.05). No significant relationship between illumination at work and MSDs of the knee in males. No significant relationship between hot environment, noise at workplace, or dusty work environment and MSDs of the knee in females and males.
Namkaew and Wiwatanadate in 2012 [63], Thailand, NR	General population, 534, NR, ≥ 50 years.	Inclusion: age ≥ 50 years, lived in Pookha or Ontai subdistrict for > 30 years, and never moved to other places. Exclusion: congenital abnormality, neurological diseases, cancer with neuropathic pain, use of artiﬁcial aids ⁄tools for extremities.	Water quality. Quantitative measure of average daily fluoride dose (ADFD) in drinking water.	LE. Thai version of 11-point Likert current pain scale (PRO).	Logistic regression. No significant relationship between ADFD and knee or leg pain.
Noormohammadpour et al. in 2017 [64], Iran, Tehran University of Medical Sciences and Health Services, Centre for Disease Control and Management	General population, 7,889 (4,745 F, 3,144 M), 50.9 ± 13.1 years.	Inclusion: resident of Iran, household in 429 districts as identified by postal code. Exclusion: NR.	Air quality and ventilation. Smoking history including passive smoking (ETS), assessed via interview (PRO).	LE. Chronic MSK knee pain > 3 months over the past year assessed with questionnaire (PRO).	Chi-square, logistic regression. Exposure to ETS (passive smoking) was associated with increased risk of chronic knee pain (OR 1.21, 95% CI 1.06-1.38, p < 0.05).
Pal et al. in 2021 [65], India, NR	Garment factory workers, 222 (66 F, 156 M), 39.9 ± 13.5 years.	Inclusion: Garment factory workers in India. Exclusion: NR.	Air quality and ventilation, lighting and views, noise, thermal health, OWE. perceived work environment, with 6 variables including lighting, noise, temperature, ventilation, assessed via interview: 6-point Likert scale (PRO).	General. MSDs assessed with Cornell Musculoskeletal Discomfort Questionnaire (PRO).	NR. Unsatisfactory work environment was significantly associated with an increased prevalence of MSDs (OR 8.38, 95% CI 1.95-36.06, p < 0.05).
Pensri et al. in 2010 [66], Thailand, Social Security Ofﬁce of Thailand	Department store sales workers, 1,200 (1,200 F, 0 M), 27.8 ± 5.6 years.	Inclusion: Woman, worked in one of 18 department stores in Bangkok, work experience of ≥ 1 year, work in standing posture for an average of ≥ 5 h/day. Exclusion: NR.	Air quality and ventilation, lighting and views, noise, thermal health. Work environment, including light intensity, noise level, temperature, and air circulation, assessed with questionnaire (PRO).	LE. Prevalence of LE MSK pain: NMQ (PRO).	Chi-square. Logistic regression. Disagreeing with the statement "Light intensity in the workplace is good" is significantly associated with a decreased risk of hip MSK symptoms (OR 0.75, 95% CI 0.57-0.99, p = 0.046). No significant relationships between noise level, temperature, air circulation, and hip MSK symptoms. Disagreeing with the statement "temperature in the workplace is not too cold nor too warm" is significantly associated with a decreased risk of knee MSK symptoms (OR 0.72, 95% CI 0.55-0.93, p = 0.012). No significant relationships between light intensity, noise level, air circulation, and knee MSK symptoms. No significant relationships between light intensity, noise level, temperature, air circulation, and ankle-foot MSK symptoms.
Piedrahita et al. in 2004 [67], Colombia, NR	Meat processing factory workers, 162 (0 F, 162 M), 18-60 years.	Inclusion: workers from 4 meat processing factories in Colombia, comprised of those exposed to cold work environment (n = 50) and not exposed to cold work environment (112). Exclusion: NR.	Thermal health. Questionnaire on cold exposure at work. Work in cold environment: yes (exposed group, workplace temperature = 2.4°C), no (control group, workplace temperature = 11.6°C).	UE, LE. MSK pain in past 12 months: NMQ (PRO).	Prevalence ratio (PR). Compared to non-exposed workers, cold-exposed workers had significantly greater prevalence of pain over the past 12 months in the shoulders (PR 3.84, 95% CI 1.61-9.17, p < 0.05), wrists/hands (PR 2.57, 95% CI 1.28-5.14, p < 0.05), and hips/thighs (PR 13.44, 95% CI 1.66-108.9, p < 0.05). No significant difference between cold-exposed and non-exposed workers in prevalence of pain in the elbows, knees, ankle/feet. No significant relationship between cold exposure and UE and LE MSK symptoms preventing workers from doing normal work.
Pinar et al. in 2013 [68], Turkey, NR	Ammunition factory workers, 955 (23 F, 932 M), 42.3 ± 6.9 years.	Inclusion: workers of ammunition section of one gun factory in Turkey. Exclusion: NR.	Thermal health. Perceived workplace environment (temperature) assessed via interview (PRO).	General. MSD symptoms for 9 body regions assessed via interview: 3 questions - “during the last 12 months, have you had a job-related ache, pain, discomfort? Or seen a physician for this complaint? Or missed any workday due to this condition?” Follow-up questions regarding MSDs (PRO).	Logistic regression, cold workplace temperature was significantly associated with increased risk of developing MSDs (OR 1.838, 95% CI 1.371-2.465, p < 0.001). No significant relationship between hot workplace temperature and MSDs.
Pisinger et al. in 2011 [69], Denmark, various research, medical, and pharmaceutical organizations in Denmark	General population, 6,784 (3,496 F, 3,288 M), 30-60 years.	Inclusion: adults in Denmark who participated in Inter99 study. Exclusion: NR.	Air quality and ventilation. ETS exposure on questionnaire: how many hours a day do you usually spend in rooms where people smoke?" (almost never, 1/2 - 1 h; 1 - 5 h; > 5 h) (PRO).	General. MSK symptoms in past 12 months, 6-item questionnaire with 4-point Likert scale, categorized to dichotomous variable yes/no (PRO).	Logistic regression. In non-smokers, exposure to ETS ≥ 5 h/day was significantly associated with increased risk of MSK pain (OR 1.46, 95% CI 1.2-1.8, p < 0.05).
Raatikka et al. in 2007 [70], Finland, Finnish Environmental Cluster Research Programme	General population, 5,320 (2,926 F, 2,394 M), 25-64 years.	Inclusion: adults in Finland who participated in the FINRISK 2002 study who completed cold questionnaire. Exclusion: over-estimation of cold exposure time.	Thermal health. 9-item questionnaire on cold exposure, thermal sensations, and cold-related symptoms (PRO).	UE, LE, general. Prevalence of cold-related symptoms from items on cold questionnaire (PRO).	Logistic regression. Increased cold exposure was significantly associated with increased risk of repeated MSK pain (female: OR 1.35, 95% CI 1.14-1.58, p < 0.05; male - OR 1.13, 95% CI 1.03-1.22, p < 0.05). For females, increased cold exposure was significantly associated with increased risk of pain in the wrists-palms (OR 1.51, 95% CI 1.18-1.91, p < 0.05), fingers (OR 1.34, 95% CI 1.11-1.60, p < 0.05), and ankles-feet (OR 1.34, 95% CI 1.08-1.64, p < 0.05). No significant relationship between cold exposure and pain in the shoulders, elbows-forearms, and knees-thighs-calves. For males, increased cold exposure was significantly associated with increased risk of pain in the shoulders (OR 1.31, 95% CI 1.12-1.51, p < 0.05), and ankles-feet (OR 1.16, 95% CI 1.03-1.30, p < 0.05). No significant relationship between cold exposure and pain in the elbows-forearms, wrists-palms, fingers, and knees-thighs-calves.
Ravibabu et al. in 2019 [71], India, Indian Council of Medical Research	Battery manufacturing plant workers, 256 (0 F, 256 M), 37.0 ± 7.0 years.	Inclusion: workers of lead battery manufacturing plant and exposed to lead with > 2 years experience. Exclusion: NR. Controls (office workers) were matched to cases on age and socioeconomic status.	Air quality and ventilation, dusts, and pests. Quantitative assessment of serum blood lead levels. Comparison of cases with non-exposed controls.	UE, LE. Prevalence of LE MSK pain: NMQ (PRO).	Chi-square. The prevalence of MSDs was greater in workers exposed to lead compared to non-exposed controls in the shoulders (exposed: 16%, controls: 5%, p = 0.014), elbows (exposed: 8%, controls: 0%, p = 0.006), wrists/hands (exposed: 10%, controls: 1%, p = 0.015), knees (exposed: 26%, controls: 15%, p = 0.054), and ankles/feet (exposed: 11%, controls: 0%, p = 0.001). No significant difference between exposed and control workers in the prevalence of MSDs of the hips/thighs.
Rocha et al. in 2005 [72], Brazil, NR	Call center workers, 108 (94 F, 13 M, 1 NR), 21-23 years (median).	Inclusion: workers at a bank call center in Brazil. Exclusion: Absent from work at time of survey for various reasons (e.g., leave)	Lighting and views, noise, thermal health. Work-related questionnaire with numerous items, such as thermal comfort, noise, illumination with categories of good/regular, bad/very bad (PRO).	UE. Work-related questionnaire with numerous items, such as presence of neck-shoulder symptoms over past 12 months dichotomous variable (yes/no) (PRO).	Logistic regression. Bad thermal comfort was significantly associated with increased risk of neck-shoulder symptoms (OR 3.06, 95% CI 1.09-8.62, p = 0.034). No significant relationship between noise or illumination and neck-shoulder symptoms. No significant relationship between thermal comfort, noise, or illumination and wrist-hand symptoms.
Saha et al. in 2016 [73], India, NR	Smelting factory workers, 180 (gender: NR), 39.1 ± 6.7 years.	Inclusion: workers in an aluminum smelting factory in India, ≥ 6 months of MSK problems (back pain or joint pain). Exclusion: known causes of MSK problems (e.g., MVA).	Water quality. work-related questionnaire and interview with numerous items, such as drinking untreated water (PRO). Quantitative assessment of urine fluoride level.	General. Work-related questionnaire and interview with numerous items, such as MSK problems (back pain / joint pain / both) (PRO).	Logistic regression. Drinking untreated drinking water was significantly associated with increased risk of MSK pain (OR 1.51, 95% CI 1.03-2.76, p = 0.044). Increased urinary fluoride level was significantly associated with increased risk of MSK pain (OR 2.71, 95% CI 1.81-3.75, p = 0.024).
Sharma and Singh in 2014 [74], India, NR	Foundry industry workers, 516 (263 F, 253 M), 37.8 ± 11.0 years.	Inclusion: foundry workers in Agra, India. Exclusion: NR.	Lighting and views, noise, thermal health. Work environment, including hot environment, noise, illumination, assessed with a questionnaire: 5-point Likert scale (PRO).	UE. Prevalence of MSDs assessed with NIOSH checklist (PRO). Pain severity assessed with a questionnaire: 4-point Likert scale (PRO).	Pearson's correlation, logistic regression. Hot environment, noise at workplace, and less illumination were significantly associated with increased prevalence of MSDs of the shoulders in certain female workers (p < 0.05). No significant relationships between hot environment, noise at workplace, or less illumination and MSDs of the shoulders in male workers.
Sormunen et al. 2009 [75], Finland, Centre for Occupational Safety	Meat processing and dairy workers, 1,117 (514 F, 603 M), 34 ± 10.5 years.	Workers in 5 meat processing factories and 2 dairies in Finland. Exclusion: NR.	Thermal health. Work-related questionnaire with numerous items, such as being exposed to uncomfortable cold in neck-shoulder or low back at work, categorized on 4-point Likert scale (PRO).	UE. Questionnaire with items on MSK pain causing disadvantage in daily routines during past 12 months, categorized as yes/no (PRO).	Logistic regression. Exposure to uncomfortable cold (slight, some, extensive) was significantly associated with increased risk of shoulder and wrist pain causing disadvantage in daily routines (shoulder, extensive cold: OR 6.32, 95% CI 2.54-15.74, p < 0.05; wrist, extensive cold: OR 21.65, 95% CI 11.58-40.46, p < 0.05).
Stjernbrandt et al. in 2018 [76], Sweden, Umea University, Vasterbotten County Council	General population, 997 (632 F, 365 M), 60-70 years (median).	Inclusion: participant of Cold and Health in Northern Sweden (CHINS) research study, resident of Norrbotten, Vasterbotten, Vasternorrland, and Jamtland counties, name available in Swedish population register, cases - "yes" answers to "I am oversensitive to cold" and "I experience pain/discomfort when fingers/hands are exposed to cold, controls - "No" answers to 2 questions above, no Raynaud's syndrome. Exclusion: NR. Controls were matched to cases on geographical area, sex, and age.	Thermal health. Severity of cold sensitivity assessed with 100 mm VAS (100 mm) and cold intolerance symptom severity score (4-100) (PRO).	UE. Presence of UE nerve injury and carpal tunnel syndrome assessed via HCP interview (PRO).	Logistic regression. Increased cold sensitivity was significantly associated with increased risk of UE nerve injury (OR 2.0, 95% CI 1.3-3.0, p < 0.05). No significant relationship between cold sensitivity and risk of carpal tunnel syndrome.
Sundstrup et al. in 2015 [77], Denmark, Danish Parliament, Danish Working Environment Research Fund	Slaughterhouse workers, 82 (0 F, 82 M), 45.0 ± 11.0 years.	Inclusion: workers with chronic upper limb pain (cases) and without chronic upper limb pain (controls) in 2 large-scale slaughterhouses, male. Exclusion: hypertension (BP > 160/100 mmHg), history of cardiovascular diseases, symptoms of carpal tunnel syndrome. Recent traumatic injury to necks shoulders, arms, or hands, pregnancy.	Air quality and ventilation, dusts, and pests, lighting and views, noise, thermal health, OWE. Prevalence of indoor environmental complaints assessed by the MM Indoor Climate Questionnaire rating bothersomeness of 13 environmental factors: often/sometimes/never (PRO).	UE. Chronic UE MSK pain group subjects (cases) defined as: 1. pain intensity in the shoulder, elbow/forearm, or hand/wrist of ≥ 3 on 0-10 VAS during past 3 months, 2. pain intensity in the shoulder, elbow/forearm, or hand/wrist of ≥ 3 on 0-10 VAS during past week, 3. pain that has lasted > 3 months, 4. frequency of pain of ≥ 3 days per week during past week, and 5. ≥ “some” work disability on a 5-point scale, “not at all,” “a little,” “some,” and “much” to “very much,” when asked “during the last 3 months, did you have any difficulty performing your work due to pain? (PRO). Control group subjects did not meet these criteria.	Chi-square, logistic regression. Chi-square. The prevalence of indoor climate complaints was significantly greater in workers with chronic UE pain compared to pain-free controls for draught (chronic pain: 40%, control: 6%, p < 0.05) and noise (chronic pain: 58%, control: 33%, p < 0.05). No significant difference between workers with chronic UE pain and pain-free controls in the prevalence of indoor climate complaints for room temperature (too high, too low, or varying), stuffy and dry air, unpleasant odor, passive smoking, lighting problems, and dust and dirt. The average number of indoor climate complaints was significantly greater for workers with chronic UE pain compared to pain-free controls (chronic pain: mean 1.8, 95% CI 1.3-2.3, control: mean 0.9, 95% CI 0.4-1.5, p = 0.0163).
Thetkathuek et al. in 2015 [78], Thailand, Burapha University and National Research Council	Frozen food processing workers, 752 (434 F, 314 M), 15-53 years.	Inclusion: individuals exposed (n = 497) and not exposed (n = 255) to cold work environments in two frozen food factories in Rayong, Thailand. Exclusion: NR.	Thermal health. Work-related interview with numerous items including cold exposure symptoms, categorized as dichotomous variable (yes/no) (PRO).	General. Work-related interview with numerous items including repeated MSK pain and symptoms (back or muscular, extremity) categorized as dichotomous variable (yes/no) (PRO).	Logistic regression. Compared to non-exposed office workers, warehouse workers exposed to cold work conditions had higher rates of general musculoskeletal symptoms (back pain, muscular pain) (exposed 89.1%, non-exposed 18.4%, p < 0.05) and extremity symptoms in the digits (fingers, toes) (exposed 89.1%, non-exposed 50.0%, p < 0.05). Exposure to cold work conditions was significantly associated with increased risk of general musculoskeletal symptoms (exposed warehouse workers: OR 11.96, 95% CI 6.12-23.45, p < 0.05) and extremity symptoms (exposed warehouse workers: OR 13.51, 95% CI 5.17-35.33, p < 0.05).
Vasseljen et al. in 2001 [79], Norway, NR	Customer relations workers, 66 (66 F, 0 M), NR.	Inclusion: female customers relations workers in healthcare and shopping center facilities in Norway, ≥ 2 years work experience in current or similar position, ≥ 50% full-time. Exclusion: Neck-shoulder pain due to injury or systemic disease, fibromyalgia, pregnant.	Air quality and ventilation, lighting and views, moisture, noise, Thermal health, OWE. Work-related questionnaire with numerous items including one item on indoor environment (air, humidity, light, noise, and temperature) assessed on 10 cm VAS (PRO).	UE. Neck-shoulder pain over past 24 h, 7 days, 6 months assessed with 6-point NRS (PRO).	T-test. Indoor environment score was worse in workers with neck-shoulder pain compared to no pain (pain: mean 3.2, 95% CI 2.5-3.9, no pain: mean 4.6, 95% CI 3.6-5.6, p = 0.02).
Vignoli et al. in 2015 [80], Italy, NR	Grocery store workers, 553 (348 F, 205 M), 43.4 ± 7.8 years.	Inclusion: grocery store workers from a large retail company in Italy. Exclusion: NR.	Safety and security. Workplace bullying assessed with Short Negative Acts Questionnaire (PRO).	UE. Shoulder MSDs over past 12 months assessed with question - “during the past 12 months have you had pain, aching, stiffness, burning, numbness, or tingling (“pins and needles”) in the (shoulder) that occurred more than three times or at least more than a week?”: yes/no (PRO).	Logistic regression. Workplace bullying was significantly associated with shoulder MSDs (R = 0.141, p < 0.01).
Waller et al. in 2019 [81], Australia, Various academic, research, and private organizations in Australia	General population, 917 (475 F, 442 M), 22.1 ± 0.7 years.	Inclusion: 22-year follow-up data available from the Western Australian Pregnancy Cohort (Raine) Study, born to women who enrolled in study before 18th gestational week, completed OMPQ, had data for ≥ 1 valid pain sensitivity test. Exclusion: NR.	Thermal health. Cold pain threshold assessed modular sensory analyzer thermal stimulator.	General. Prevalence of MSK pain assessed with OMPQ (PRO).	Tobit regression. Linear regression. Compared to those without MSK pain, participants with medium and high MSK pain had increased cold pain sensitivity (medium pain: regression coefficient 2.1, 95% CI 0.1-4.2; high pain: regression coefficient 2.3, 95% CI 0.1-4.4; p = 0.023).
Yang et al. in 2019 [82], China, no extramural funding	Intensive care unit nurses, 679 (601 F, 78 M), 28.2 ± 4.5 years.	Inclusion: registered nurses working in intensive care units in Hunan Province of China, engaged in patient care in daily work, ≥ 1 of work experience. Exclusion: only performed administrative work.	Safety and security. Perception of workplace safety and environment assessed with Hospital Safety Climate Questionnaire (PRO).	General. Prevalence of MSK pain: NMQ (PRO).	Logistic regression. Safety environment score was significantly worse for participants with MSDs compared to those without MSDs (with MSDs: 62.84 ± 15.54, without MSDs: 47.15 ± 17.19, p < 0.001). Lack of a safe work environment was significantly associated with increased risk of MSDs (OR 1.06, 95% CI 1.02-1.10, p = 0.002).
Zhang et al. in 2016 [83], China, National Natural Science Foundation of China, Department of Health of Shanxi Province	General population, 7,126 (3,517 F, 3,609 M), ± years.	Inclusion: permanent residents of specified villages in Shanxi province of China, ages ≥ 16 years, evaluated within the Community Oriented Program for the Control of Rheumatic Diseases of the World Health Organization. Exclusion: NR.	Air quality and ventilation, thermal health. Ventilation at home assessed via NR method and categorized as poor, average, better than average. Heating at home assessed via NR method and categorized as None, adobe bed, Stove, heating installation.	General. Presence of osteoarthritis was defined by American College of Rheumatology criteria and radiographic evidence of osteoarthritis defined as Kellgren-Lawrence grade ≥ 2.	Chi-square, logistic regression. Poor ventilation at home was significantly associated with increased incidence of osteoarthritis (poor: incidence rate 41.9%, better than average 22.9%, p < 0.001). Poor heating at home was significantly associated with increased incidence of osteoarthritis (poor (none): incidence rate 33.5%, good (installed heating) 17.0%, p < 0.001).
Zimmerman et al. in 2020 [84], Sweden, Lund University, Skane University Hospital, Malmo Diabetes Association, Swedish Diabetes Foundation	Individuals from general population with carpal tunnel syndrome who had surgery, 3,438 (2,257 F, 1,181 M), 56.0 ± 16.0 years.	Inclusion: data available from the Swedish National Registry for Hand Surgery, ages ≥ 18 years, open carpal tunnel release surgery between 2010-2016. Exclusion: NR.	Thermal health. Cold sensitivity assessed with HAKIR questionnaire 8: mild/moderate/severe (PRO).	UE. Disability related to carpal tunnel syndrome assessed with the Quick Disabilities of the Arm Shoulder and Hand Questionnaire (QuickDASH) (PRO).	Kruskal-Wallis, Chi-square. Moderate and severe cold sensitivity was significantly associated with increased disability (as assessed by QuickDASH) related to carpal tunnel syndrome at pre-op, 3-month post-op, and 12-month post-op (pre-op - mild: mean 40, IQR 25-55; moderate: mean 53, IQR 39-66, severe: mean 64, IQR 50-75, p < 0.0001; 3-month - mild: mean 18, IQR 9-32; moderate: mean 20, IQR 9-39, severe: mean 32, IQR 14-52, p < 0.0001; 12-month - mild: mean 9, IQR 2-23; moderate: mean 18, IQR 3-37, severe: mean 25, IQR 7-50, p < 0.0001).
Zwart et al. in 1998 [85], Norway, NR	Individuals from general population with unilateral sciatica, 40 (17 F, 23 M), 42.7 ± 10.4 years.	Inclusion: individuals with L5 or S1 unilateral sciatica, being evaluated for lumbar disc surgery. Exclusion: other neurological disorders, diabetes, vascular claudication.	Thermal health. Warm and cold sensory thresholds assessed via Somedic thermotest. Comparison within subject side-to-side.	LE. 5 and S1 sciatica (radiculopathy) assessed via clinical and radiological examinations.	ANOVA, Chi-square, T-test. In all patients, warm sensory threshold was significantly worse on the symptomatic side compared to the asymptomatic side (symptomatic: 8.4 ± 3.0, asymptomatic: 6.2 ± 2.5, p < 0.0005). No significant difference in cold sensory threshold between symptomatic and asymptomatic sides. In operated patients with confirmed disc prolapse, warm sensory threshold was significantly worse on the symptomatic side compared to the asymptomatic side (symptomatic: 8.2 ± 3.1, asymptomatic: 5.9 ± 2.6, p < 0.0005). Cold sensory threshold was significantly worse on the symptomatic side compared to the asymptomatic side (symptomatic: 2.1 ± 1.6, asymptomatic: 1.5 ± 0.9, p < 0.003).

**Table 4:** Type and quality of included studies. CD: cannot determine; NA: not applicable; N: no; NR: not reported; Y: yes; Overall quality rating: 0-4 = poor (high risk of bias); 5-9 = fair (between low and high risk of bias); 10-14 = good (low risk of bias). Study quality determined by the NIH quality assessment tool [32], with 14 items: 1. was the research question or objective in this paper clearly stated? 2. Was the study population clearly specified and defined? 3. Was the participation rate of eligible persons at least 50%? 4. Were all the subjects selected or recruited from the same or similar populations (including the same time period)? Were inclusion and exclusion criteria for being in the study prespecified and applied uniformly to all participants? 5. Was a sample size justification, power description, or variance and effect estimates provided? 6. For the analyses in this paper, were the exposure(s) of interest measured prior to the outcome(s) being measured? 7. Was the timeframe sufficient so that one could reasonably expect to see an association between exposure and outcome if it existed? 8. For exposures that can vary in amount or level, did the study examine different levels of the exposure as related to the outcome (e.g., categories of exposure, or exposure measured as continuous variable)? 9. Were the exposure measures (independent variables) clearly defined, valid, reliable, and implemented consistently across all study participants? 10. Was the exposure(s) assessed more than once over time? 11. Were the outcome measures (dependent variables) clearly defined, valid, reliable, and implemented consistently across all study participants? 12. Were the outcome assessors blinded to the exposure status of participants? 13. Was loss to follow-up after baseline 20% or less? 14. Were key potential confounding variables measured and adjusted statistically for their impact on the relationship between exposure(s) and outcome(s)?

Author, year	Study type (level of evidence)	Q1	Q2	Q3	Q4	Q5	Q6	Q7	Q8	Q9	Q10	Q11	Q12	Q13	Q14	Overall quality score	Overall quality rating
Ahmad et al. in 2014 [33]	Cross-sectional (4)	Y	Y	CD	Y	N	N	N	Y	Y	N	N	NR	NA	N	5	Fair
Alhusuny et al. 2021 [34]	Cross-sectional (4)	Y	Y	CD	Y	N	N	N	Y	Y	N	Y	NR	NA	Y	7	Fair
Assunção and Abreu in 2017 [35]	Cross-sectional (4)	Y	Y	CD	Y	N	N	N	N	Y	N	Y	NR	NA	Y	6	Fair
Bang et al. in 2005 [36]	Cross-sectional (4)	Y	Y	N	Y	N	N	N	N	Y	N	Y	NR	NA	N	5	Fair
Barro et al. in 2015 [37]	Cross-sectional (4)	Y	Y	CD	Y	N	N	N	Y	Y	N	Y	NR	NA	Y	7	Fair
Carnow and Conibear in 1981 [38]	Cross-sectional (4)	Y	N	CD	CD	N	N	N	Y	Y	N	N	NR	NA	N	3	Poor
Coombes et al. in 2015 [39]	Prospective cohort (2)	Y	Y	CD	Y	N	Y	Y	Y	Y	Y	Y	NR	Y	Y	11	Good
Coronado et al. in 2011 [40]	Cross-sectional (4)	Y	Y	CD	Y	N	N	N	Y	Y	N	Y	Y	NA	Y	8	Fair
Coronado et al. in 2014 [41]	Case-control (3)	Y	Y	CD	Y	N	N	N	Y	Y	N	Y	NR	NA	Y	7	Fair
Coronado et al. in 2014 [42]	Prospective cohort (2)	Y	Y	CD	Y	Y	Y	Y	Y	Y	Y	Y	NR	Y	Y	12	Good
Dahl et al. in 2014 [43]	Prospective cohort (2)	Y	Y	CD	Y	N	Y	Y	Y	Y	Y	Y	NR	CD	Y	10	Good
Dahl et al. in 2013 [44]	Prospective cohort (2)	Y	Y	Y	Y	N	Y	Y	Y	Y	Y	Y	NR	CD	Y	11	Good
d'Errico et al. in 2010 [45]	Cross-sectional (4)	Y	Y	CD	Y	N	N	N	Y	Y	N	Y	NR	NA	Y	7	Fair
Douphrate et al. in 2016 [46]	Cross-sectional (4)	Y	Y	CD	Y	N	N	N	Y	Y	N	Y	NR	NA	Y	7	Fair
Farbu et al. in 2019 [47]	Cross-sectional (4)	Y	Y	Y	Y	N	N	N	Y	Y	N	Y	NR	NA	Y	8	Fair
Ghani et al. in 2020 [48]	Cross-sectional (4)	Y	Y	CD	Y	N	N	N	Y	Y	N	Y	NR	NA	N	6	Fair
Ignatius et al. in 1993 [49]	Cross-sectional (4)	Y	N	Y	CD	N	N	N	N	Y	N	N	NR	NA	N	3	Poor
Inaba et al. in 2011 [50]	Cross-sectional (4)	Y	Y	CD	CD	N	N	N	Y	Y	N	Y	NR	NA	N	5	Fair
Janwantanakul et al. in 2010 [51]	Cross-sectional (4)	Y	Y	Y	Y	N	N	N	N	Y	N	Y	NR	NA	Y	7	Fair
Jensen et al. in 2019 [52]	Cross-sectional (4)	Y	Y	Y	Y	N	N	N	Y	Y	N	Y	NR	NA	Y	8	Fair
Kang et al. in 2016 [53]	Cross-sectional (4)	Y	Y	Y	Y	N	N	N	Y	Y	N	Y	NR	NA	Y	8	Fair
Kaufman-Cohen et al. in 2011 [54]	Cross-sectional (4)	Y	Y	N	Y	N	N	N	CD	Y	N	Y	NR	NA	Y	6	Fair
Kesavachandran et al. in 2009 [55]	Case-control (3)	Y	Y	CD	Y	N	N	N	Y	Y	N	Y	NR	NA	N	6	Fair
Kurttio et al. in 1999 [56]	Retrospective cohort (3)	Y	Y	CD	Y	N	Y	Y	Y	Y	Y	Y	NR	CD	Y	10	Good
Magnavita et al. in 2011 [57]	Cross-sectional (4)	Y	Y	Y	Y	N	N	N	Y	Y	N	Y	NR	NA	Y	8	Fair
Mekonnen et al. in 2020 [58]	Cross-sectional (4)	Y	Y	Y	Y	Y	N	N	N	Y	N	Y	NR	NA	Y	8	Fair
Miettinen et al. in 2021 [59]	Prospective cohort (2)	Y	Y	Y	Y	N	Y	Y	Y	Y	Y	Y	NR	N	Y	11	Good
Miranda et al. in 2011 [60]	Prospective cohort (2)	Y	Y	N	Y	N	Y	Y	Y	Y	Y	Y	NR	CD	Y	10	Good
Nag et al. 2015 [61]	Cross-sectional (4)	Y	Y	Y	Y	N	N	N	Y	Y	N	Y	NR	NA	Y	8	Fair
Nag et al. in 2010 [62]	Cross-sectional (4)	Y	Y	Y	Y	N	N	N	Y	Y	N	Y	NR	NA	Y	8	Fair
Namkaew and Wiwatanadate in 2012 [63]	Cross-sectional (4)	Y	Y	CD	Y	N	N	N	Y	Y	N	Y	NR	NA	Y	7	Fair
Noormohammadpour et al. in 2017 [64]	Cross-sectional (4)	Y	Y	CD	Y	N	N	N	CD	Y	N	Y	NR	NA	Y	6	Fair
Pal et al. in 2021 [65]	Cross-sectional (4)	Y	Y	CD	Y	N	N	N	N	Y	N	Y	NR	NA	Y	6	Fair
Pensri et al. in 2010 [66]	Cross-sectional (4)	Y	Y	Y	Y	N	N	N	N	Y	N	Y	NR	NA	Y	7	Fair
Piedrahita et al. in 2004 [67]	Cross-sectional (4)	Y	Y	Y	CD	N	N	N	N	Y	N	Y	NR	NA	Y	6	Fair
Pinar et al. in 2013 [68]	Cross-sectional (4)	Y	Y	Y	Y	N	N	N	Y	Y	N	Y	NR	NA	Y	8	Fair
Pisinger et al. in 2011 [69]	Cross-sectional (4)	Y	Y	CD	Y	N	N	N	N	Y	N	Y	NR	NA	Y	6	Fair
Raatikka et al. in 2007 [70]	Cross-sectional (4)	Y	Y	Y	Y	N	N	N	Y	Y	N	Y	NR	NA	Y	8	Fair
Ravibabu et al. in 2019 [71]	Case-control (3)	Y	Y	CD	N	N	N	N	Y	Y	N	N	Y	NA	N	5	Fair
Rocha et al. in 2005 [72]	Cross-sectional (4)	Y	Y	Y	Y	N	N	N	Y	Y	N	Y	NR	NA	Y	8	Fair
Saha et al. in 2016 [73]	Cross-sectional (4)	Y	Y	Y	Y	Y	N	N	Y	Y	N	Y	NR	NA	Y	9	Fair
Sharma and Singh in 2014 [74]	Cross-sectional (4)	Y	Y	CD	Y	N	N	N	Y	Y	N	Y	NR	NA	Y	7	Fair
Sormunen et al. in 2009 [75]	Cross-sectional (4)	Y	Y	Y	Y	N	N	N	Y	Y	N	Y	NR	NA	Y	8	Fair
Stjernbrandt et al. in 2018 [76]	Case-control (3)	Y	Y	N	Y	N	N	N	Y	Y	N	Y	NR	NA	Y	7	Fair
Sundstrup et al. in 2015 [77]	Case-control (3)	Y	Y	CD	Y	N	N	N	Y	Y	N	Y	Y	NA	Y	8	Fair
Thetkathuek et al. in 2015 [78]	Cross-sectional (4)	Y	Y	CD	Y	Y	N	N	Y	Y	N	Y	NR	NA	Y	8	Fair
Vasseljen et al. in 2001 [79]	Cross-sectional (4)	Y	Y	Y	Y	N	N	N	Y	Y	N	Y	NR	NA	Y	8	Fair
Vignoli et al. in 2015 [80]	Cross-sectional (4)	Y	Y	Y	Y	N	N	N	Y	Y	N	Y	NR	NA	Y	8	Fair
Waller et al. in 2019 [81]	Cross-sectional (4)	Y	Y	CD	Y	N	N	N	Y	Y	N	Y	NR	NA	Y	7	Fair
Yang et al. in 2019 [82]	Cross-sectional (4)	Y	Y	Y	Y	N	N	N	Y	Y	N	Y	NR	NA	Y	8	Fair
Zhang et al. in 2016 [83]	Case-control (3)	Y	Y	CD	Y	N	N	N	Y	Y	N	Y	NR	NA	Y	7	Fair
Zimmerman et al. in 2020 [84]	Prospective cohort (2)	Y	Y	CD	Y	N	Y	Y	Y	Y	Y	Y	NR	CD	Y	10	Good
Zwart et al. in 1998 [85]	Cross-sectional (4)	Y	Y	CD	Y	N	N	N	Y	Y	N	Y	NR	NA	N	6	Fair

**Table 5:** PRISMA 2020 checklist. PRISMA: Preferred Reporting Items for Systematic Reviews and Meta-Analyses

Section and topic	Item #	Checklist item	Location where the item is reported
Title: Relationship of Healthy Building Determinants With Musculoskeletal Disorders of the Extremities: A Systematic Review			Page number from manuscript
Title	1	Identify the report as a systematic review.	1
Abstract			
Abstract	2	See the PRISMA 2020 for Abstracts checklist.	1
Introduction			
Rationale	3	Describe the rationale for the review in the context of existing knowledge.	1, 2
Objectives	4	Provide an explicit statement of the objective(s) or question(s) the review addresses.	2
Methods			
Eligibility criteria	5	Specify the inclusion and exclusion criteria for the review and how studies were grouped for the syntheses.	2, 3
Information sources	6	Specify all databases, registers, websites, organizations, reference lists, and other sources searched or consulted to identify studies. Specify the date when each source was last searched or consulted.	2, 3
Search strategy	7	Present the full search strategies for all databases, registers, and websites, including any filters and limits used.	2, Table 2
Selection process	8	Specify the methods used to decide whether a study met the inclusion criteria of the review, including how many reviewers screened each record and each report retrieved, whether they worked independently, and if applicable, details of automation tools used in the process.	3
Data collection process	9	Specify the methods used to collect data from reports, including how many reviewers collected data from each report, whether they worked independently, any processes for obtaining or confirming data from study investigators, and if applicable, details of automation tools used in the process.	3
Data items	10a	List and define all outcomes for which data were sought. Specify whether all results that were compatible with each outcome domain in each study were sought (e.g., for all measures, time points, analyses), and if not, the methods used to decide which results to collect.	3
	10b	List and define all other variables for which data were sought (e.g. participant and intervention characteristics, funding sources). Describe any assumptions made about any missing or unclear information.	3
Study risk of bias assessment	11	Specify the methods used to assess risk of bias in the included studies, including details of the tool(s) used, how many reviewers assessed each study and whether they worked independently, and if applicable, details of automation tools used in the process.	4
Effect measures	12	Specify for each outcome the effect measure(s) (e.g. risk ratio, mean difference) used in the synthesis or presentation of results.	3, 4
Synthesis methods	13a	Describe the processes used to decide which studies were eligible for each synthesis (e.g. tabulating the study intervention characteristics and comparing against the planned groups for each synthesis (item #5)).	3, 4
	13b	Describe any methods required to prepare the data for presentation or synthesis, such as handling of missing summary statistics, or data conversions.	3, 4
	13c	Describe any methods used to tabulate or visually display results of individual studies and syntheses.	3, 4, Table 1, Supplementary Table 3
	13d	Describe any methods used to synthesize results and provide a rationale for the choice(s). If meta-analysis was performed, describe the model(s), method(s) to identify the presence and extent of statistical heterogeneity, and software package(s) used.	4
	13e	Describe any methods used to explore possible causes of heterogeneity among study results (e.g. subgroup analysis, meta-regression).	not applicable as noted on page 4
	13f	Describe any sensitivity analyses conducted to assess robustness of the synthesized results.	not applicable as noted on page 4
Reporting bias assessment	14	Describe any methods used to assess risk of bias due to missing results in a synthesis (arising from reporting biases).	not applicable as noted on page 4
Certainty assessment	15	Describe any methods used to assess certainty (or confidence) in the body of evidence for an outcome.	4
Results			
Study selection	16a	Describe the results of the search and selection process, from the number of records identified in the search to the number of studies included in the review, ideally using a flow diagram.	4, 5, Figure 1
	16b	Cite studies that might appear to meet the inclusion criteria, but which were excluded, and explain why they were excluded.	5, Figure 1
Study characteristics	17	Cite each included study and present its characteristics.	5, 6 Table 3
Risk of bias in studies	18	Present assessments of risk of bias for each included study.	6, Table 2
Results of individual studies	19	For all outcomes, present, for each study: (a) summary statistics for each group (where appropriate) and (b) an effect estimate and its precision (e.g. confidence/credible interval), ideally using structured tables or plots.	6, 7, 8 Table 3
Results of syntheses	20a	For each synthesis, briefly summarise the characteristics and risk of bias among contributing studies.	4, 5, 6, 7, 8 Table 1, Tables 3, 4
	20b	Present results of all statistical syntheses conducted. If meta-analysis was done, present for each the summary estimate and its precision (e.g. confidence/credible interval) and measures of statistical heterogeneity. If comparing groups, describe the direction of the effect.	not applicable as noted on page 4
	20c	Present results of all investigations of possible causes of heterogeneity among study results.	not applicable as noted on page 4
	20d	Present results of all sensitivity analyses conducted to assess the robustness of the synthesized results.	not applicable as noted on page 4
Reporting biases	21	Present assessments of risk of bias due to missing results (arising from reporting biases) for each synthesis assessed.	not applicable as noted on page 4
Certainty of evidence	22	Present assessments of certainty (or confidence) in the body of evidence for each outcome assessed.	6, 7, 8, Table 1
Discussion			
Discussion	23a	Provide a general interpretation of the results in the context of other evidence.	8, 9
	23b	Discuss any limitations of the evidence included in the review.	9
	23c	Discuss any limitations of the review processes used.	9
	23d	Discuss implications of the results for practice, policy, and future research.	9
Other information			
Registration and protocol	24a	Provide registration information for the review, including register name and registration number, or state that the review was not registered.	1, 2
	24b	Indicate where the review protocol can be accessed, or state that a protocol was not prepared.	1, 2 (PROSPERO registration)
	24c	Describe and explain any amendments to information provided at registration or in the protocol.	not applicable
Support	25	Describe sources of financial or non-financial support for the review, and the role of the funders or sponsors in the review.	27
Competing interests	26	Declare any competing interests of review authors.	27
Availability of data, code, and other materials	27	Report which of the following are publicly available and where they can be found: template data collection forms; data extracted from included studies; data used for all analyses; analytic code; any other materials used in the review.	28
